# Applications of Technological Solutions in Primary Ways of Preventing Transmission of Respiratory Infectious Diseases—A Systematic Literature Review

**DOI:** 10.3390/ijerph182010765

**Published:** 2021-10-14

**Authors:** Gleidson Sobreira Leite, Adriano Bessa Albuquerque, Plácido Rogerio Pinheiro

**Affiliations:** UNIFOR, Department of Computer Science, University of Fortaleza, Fortaleza 60811-905, Ceará, Brazil; adrianoba@unifor.br (A.B.A.); placido@unifor.br (P.R.P.)

**Keywords:** healthcare, infectious disease, information technology, literature review, transmission prevention

## Abstract

With the growing concern about the spread of new respiratory infectious diseases, several studies involving the application of technology in the prevention of these diseases have been carried out. Among these studies, it is worth highlighting the importance of those focused on the primary forms of prevention, such as social distancing, mask usage, quarantine, among others. This importance arises because, from the emergence of a new disease to the production of immunizers, preventive actions must be taken to reduce contamination and fatalities rates. Despite the considerable number of studies, no records of works aimed at the identification, registration, selection, and rigorous analysis and synthesis of the literature were found. For this purpose, this paper presents a systematic review of the literature on the application of technological solutions in the primary ways of respiratory infectious diseases transmission prevention. From the 1139 initially retrieved, 219 papers were selected for data extraction, analysis, and synthesis according to predefined inclusion and exclusion criteria. Results enabled the identification of a general categorization of application domains, as well as mapping of the adopted support mechanisms. Findings showed a greater trend in studies related to pandemic planning and, among the support mechanisms adopted, data and mathematical application-related solutions received greater attention. Topics for further research and improvement were also identified such as the need for a better description of data analysis and evidence.

## 1. Introduction

According to Baldominus et al. [1], infectious diseases are the result of the invasive action of microscopic organisms (e.g., bacteria or viruses) in the body, and may be presented in many different types with different effect ranges. For example, while some infected bodies can remain asymptomatic, others can reach high risks of death.

Given the importance of the issue and its impact on human life, medicine has developed a variety of mechanisms for the prevention, prediction, diagnosing and treatment of infections [1]. However, over the years, new microorganisms have emerged, generating constant challenges for science in combating the action of these organisms towards humanity.

An important example of this scenario is the current pandemic crisis brought about by the new coronavirus (SARS-CoV-2), responsible for COVID-19 disease. Discovered in Wuhan and rapidly spread since December 2019 within China to other countries of the world [2,3], the newly identified coronavirus has generated considerable challenges both in terms of safety in public health, as well as economic and social impacts to society [4].

Due to the high occurrence rate, as well as severe health symptoms and high fatalities worldwide, on 31 January 2020, the World Health Organization (WHO) announced a global pandemic and on 11th March, the COVID-19 disease was recognized as a pandemic [5].

Since then, the number of cases and fatalities have been constantly making headlines around the world, where on 9 August 2021, the number of confirmed COVID-19 cases reached over 203 million with more than 213 countries and regions affected by the pandemic [6,7].

**Figure 1:**
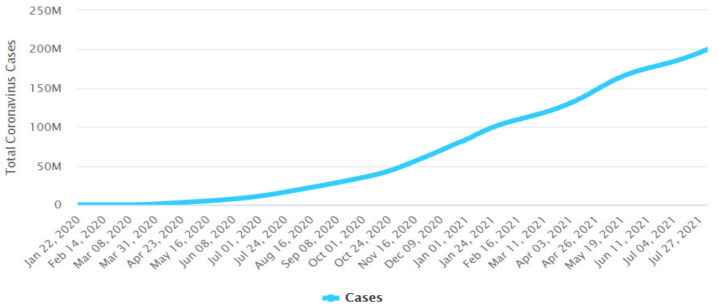
Total cumulative count of coronavirus cases (199,560,514) worldwide. Period: 22 January 2020 to 1 August 2021. Publication date: 2 August 2021. Source: https://www.worldometers.info/coronavirus/worldwide-graphs/#case-distribution (accessed on 4 August 2021).

**Figure 2:**
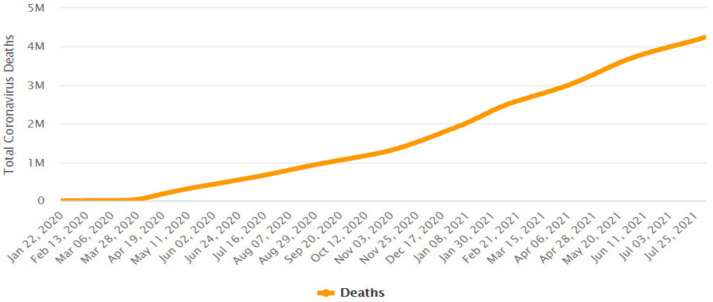
Total cumulative count of deaths caused by coronavirus (4,240,374) worldwide. Period: 23 January 2020 to 1 August 2021. Publication date: 2 August 2021. Source: https://www.worldometers.info/coronavirus/worldwide-graphs/#case-distribution (accessed on 4 August 2021).

Figure 1 presents the evolution of the total cumulative count of identified COVID-19 cases around the world in the period of 22 January 2020 to 1 August 2021, and Figure 2 presents the evolution of the total number of deaths around the world in the period of 23 January 2020 to 1August 2021.

From the emergence of new infectious diseases, new research studies are also being carried out in order to contribute to their treatment motivated not only because of health crisis, but also social and economic impacts. However, until new medications or vaccines are produced, preventive measures are recommended by health organizations in order to reduce transmission among the population, such as social distancing, mask usage, isolation and quarantine [8,9,10].

Being a topic of considerable importance, especially due to the social, health and economic impacts to society, studies focused on the application of technology in the primary forms of prevention of new infectious diseases have attracted much attention and concern from institutions and researchers.

Despite the existence of several publications presenting approaches and different uses of technology in this context, to the best of our knowledge, there are no records of research aimed at the identification, registration, selection, and rigorous analysis and synthesis of this literature.

Additionally, due to the large volume of studies, and the fact that they are published in several conferences and journals, it is difficult to locate these works.

To assist current and future researchers in discovering these studies, as well as to identify, select, rigorously analyze, and synthesize this literature, a systematic literature review (SLR) is presented in this paper.

The scope of this SLR was to identify relevant studies that adopt information technology solutions in the primary ways of preventing respiratory infectious diseases transmission/spread.

This study also aims to assist in understanding what is being carried out and studied, discovering new directions, as well as having a better understanding of how technologies are being used in the context proposed in this work, its main objectives, support mechanisms adopted, level of evidence reported, gaps that need to be deepened in research, as well as to organize the knowledge to support the technological transition.

In this paper, the design, execution and findings of a systematic review of the literature are presented, aiming at a systematic identification, selection and summarization of a comprehensive set of approaches that adopt technological solutions in the primary ways of respiratory infectious diseases transmission prevention.

In this review, 219 relevant papers (from 1139 initially retrieved) were selected and rigorously analyzed performing data extraction, analysis, and synthesis according to predefined inclusion and exclusion criteria, in order to answer a set of research questions that motivated this review.

The SLR took place in three stages. In the first one, a proposed search string (see Section 3.2.2) was executed in four digital libraries obtaining 1139 papers. In the second stage, an initial filter was performed in the title, keywords and abstract of the studies, applying the inclusion and exclusion criteria (see Section 3.3) resulting in 239 articles. Finally, performing a full text reading of the remaining articles, the inclusion and exclusion criteria were applied again, resulting in the 219 relevant studies.

From the findings, it was possible to identify six application domain categories in which there was a greater trend in studies related to pandemic planning and, among the support mechanisms adopted, data and mathematical application-related solutions received greater attention.

The two significant contributions of this study are:

- This work presents the design, execution, and results of a comprehensive systematic literature review of relevant studies on information technology applications in the primary ways of new respiratory infectious diseases transmission prevention. The study was based on predefined selection criteria, where rigorous analysis and synthesis of the approaches and associated support mechanisms were carried out, reporting the evidence in an easily accessible format.
- In this study, the approaches, support mechanisms and available evidence were structured and classified using different formats that are expected to be useful to practitioners and researchers. It is also expected that the findings will also identify issues relevant to interested researchers, and that can be used as an evidence-based guide to select appropriate technologies, approaches, solutions, or support mechanisms based on the different needs or scenarios.

## 2. Background and Related Work

Having an important role in several actions in support of treatment, combat and prevention of new infectious diseases, the adoption of information technology in this scenario has attracted great attention and concern from researchers and practitioners.

However, concerning new infectious diseases that may emerge, until the production of immunizers or medications that will make their treatment possible, preventive actions must be taken by the population to reduce the rates of contamination and fatalities, such as social distancing, mask usage, isolation, or quarantine [8,9,10].

To this end, several research studies have been proposed and carried out regarding the application of technology in this context, as mentioned by Chen et al. [11], who conducted a review on the developments and challenges of current contact tracing technologies. According to Chen et al. [11], contact tracking is one of the key technologies that may be adopted in the prevention and control of infectious diseases, which may be helpful in the location and isolation of infected people and high-risk individuals, preventing further spread of the diseases.

Also, in the line of contact tracing study, Ahmed et al. [12] presented an overview of proposed tracing app examples adopted in the fight against COVID-19, discussing the concerns reported by users regarding their usage, and outlining potential research directions for next-generation app design. Regarding challenges and future directions of contact tracing in the assistance of the fight against coronavirus, Chowdhury et al. [13] reviewed data-driven solutions and apps to identify their strength and weakness, and Hasaini et al. [14] presented a literature review of contact tracing approaches and applications adopted in governments around the world to monitor and control the spread of the COVID-19 disease.

According to Ricci et al. [15], several blockchain studies are also emerging in order to assist in the fight against COVID-19. In a survey conducted by the author, ways in which blockchain technology can be useful in supporting health actions were presented, including in contact tracing and vaccine support.

Regarding machine learning applications, Chamola et al. [16] provided a review of several machine learning algorithms that can be adopted in disaster and pandemic management, also presenting a tutorial on machine learning algorithms. Mathematical models, including compartment, statistical and machine learning models for COVID-19 transmission and diagnosis were also reviewed in a work presented by John et al. [17].

Other researches related to data science are also emerging in terms of applications in the prevention of infectious diseases. Regarding Big data, Sudana et al. [18] performed an analysis of the use of Big Data in the health domain in order to identify the benefits of its application in preventing the spread of infectious diseases such as Tuberculosis. A survey of the state of the art of researches based on data science process application for Dengue infection combat was also carried out by Siriyasatien et al. [19]. The work presented some issues to be explored and analyzed such as data sources, data preparation and representation techniques, and forecasting models.

Deep learning-based techniques were analyzed in a survey performed by Ikram et al. [20], which are classical techniques that can be used to detect COVID-19 Standard Operating Procedures (SOP), such as wearing masks or social distancing, were explored.

Studies that adopt Internet of Things Technologies have also been gaining space in actions applied in the health domain and prevention of infectious diseases. In a review carried out by Manavi et al. [21], the authors explored various Internet of Things technologies and applications adopted in contact tracing, screening, and surveillance, aiming to provide an overall understanding of the identified solutions in the fight against COVID-19.

Regarding Computational Intelligence applications, Baldominos et al. [1] performed a systematic literature review aiming to find studies that adopted computational intelligence to predict infections in patients using physiological data as features. The study analyzed 101 relevant documents between the period of 2003 and 2019, showed that automatic diagnosis of these diseases is well documented in medical literature and concluded that sepsis, Clostridium difficile infection and surgical site infections were the most addressed. Most of the identified studies adopted machine learning techniques.

Focusing on wearable devices, unobtrusive sensing systems and telehealth technologies, Ding et al. [22] presented a review on technologies and systems with various application scenarios for handling the COVID-19, and, regarding applications (apps and systems) developed by Government Institutions, Private Firms, and Individual Citizens across the world, Gupta et al. [23] performed a survey encompassing more than 100 apps to identify the different categories where technology is being used for decision making. The major areas of application covered in the study were Contact Tracing, Social Distancing, Mask Detection, Information Searching, Big Data, among others.

Regarding digital interventions for fighting COVID-19, Nazrul and Najmul Islam [24] performed a review to compare the Bangladeshi perspective with other countries. As the topic was still emerging and there was not much academic literature available, the authors reviewed online content using Google and Yahoo search engines. A total of 57 online e-resources were found, including news and blogs articles, web contents of organizations and online press releases. After identifying some digital interventions in the fight against COVID-19, both in different parts of the world and in Bangladesh, a comparative analysis was carried out and areas were proposed where Bangladesh could focus to strengthen the fight against COVID-19.

Also considering applications of technology in the COVID-19 pandemic scenario, Whitelaw et al. [25] made available a viewpoint with a framework for the application of digital technologies in pandemic management and response. The authors highlighted successful technologies applications in different countries regarding pandemic planning, surveillance, quarantine, health care, contact tracing and testing. 

Thus, over time, several surveys and reviews focused on technical solutions to provide assistance in the fight against infectious diseases have appeared in conferences or journals. However, to the best of our knowledge, there were no records of works aimed at systematic reviewing (identifying, selecting, rigorously analyzing and synthesizing) the literature focusing on the application of technological solutions in the primary ways of respiratory infectious diseases transmission prevention.

Through this systematic literature review, we are interested in finding out what technological solutions and support mechanisms are available in scientific works, and how they can contribute to the primary forms of prevention of the spread of new respiratory infectious diseases.

This work is structured as follows: in Section 3, the systematic literature review method is described and the review protocol is defined. Demographic information, quality assessment and research questions analysis are presented in Section 4. Threats to the validity, implications and limitations are discussed in Section 5 and, finally, conclusions are presented in Section 6.

## 3. Research Method

Being one of the most widely used research methods in Evidence-Based Software Engineering (EBSE), the Systematic Literature Review (SLR) provides a well-defined process for identifying, evaluating, and interpreting all available evidence relevant to a specific research question or topic, as well as evaluates existing studies on a specific phenomenon in a fair and credible manner [26].

This study was performed following the guidelines of Kitchenhamet et al. [26] involving three main phases: definition of a review protocol, conduction of the review and the review report. The adopted review protocol consists of the following elements: (i) research questions, (ii) search strategy, (iii) inclusion and exclusion criteria, (iv) study selection, (v) evaluation of study quality and (vi) data extraction and synthesis, which will be discussed in the following subsections.

### 3.1. Research Questions

Through this systematic literature review, the aim is to summarize and provide an overview of current research on “which approaches that adopted information technology in the primary ways of prevention of respiratory infectious diseases were reported in the peer-reviewed literature?”. For this, a set of research questions (RQs) were formulated (see Table 1) in order to be answered through this SLR.

The questions were defined in order to cover the objectives of this SLR, which are: identifying what approaches have been adopted or suggested in the primary ways of preventing respiratory infectious diseases using information technology solutions (RQ1), identification of the application domains of the approaches (RQ2), identification of which support mechanisms are proposed or used (RQ3), how much evidence to support the adoption of the approaches is available (RQ4), and the addressed contexts (RQ5). 

The answers to these questions can provide systematic insight and overview to researchers and practitioners regarding approaches and support mechanisms proposed in scientific studies, also helping to identify missing gaps and opportunities for improvement.

### 3.2. Search Strategy

According to Kitchenhamet et al. [26], in order to help researchers to get as many relevant studies as possible, the search strategy is essential. In this SLR, the research was conducted with various combinations of derivative terms related to the subject of the study, where the adopted search strategy was composed of the following elements: search method, search items and data sources.

#### 3.2.1. Search Method

For the search strategy, automatic searches on electronic database engines or digital libraries (listed in Table 2) were performed using the search terms presented in Section 3.2.2.

#### 3.2.2. Search Terms

The search terms adopted in this study, which were used to match paper titles, keywords and abstracts in the performed automatic search, followed the guidelines proposed Kitchenhamet et al. [26]. To define the most relevant search terms for the search, the following strategies were performed:

- Definition of terms from research questions and study topics
- Identification of synonyms, plurals and related terms
- Adoption of the logical operator “OR” to incorporate synonyms
- Concatenate parameters using the logical operator “AND”
- Check the terms in the titles of papers, keywords and abstracts

The resulted search string is composed of synonyms and terms related to “infectious diseases” AND “transmission” AND “prevention” AND “technology”, as presented as follows:

(“infectious disease” OR “infectious diseases” OR “COVID” OR “COVID-19” OR “SARS-CoV-2”) AND (“spread” OR “transmission” OR “propagation”) AND (“prevention” OR “prevent”) AND (“technology” OR “information technology”)

The terms related to “COVID-19” were also added since the inclusion of these terms was also of interest to the research. 

#### 3.2.3. Data Sources

As presented in Table 2, four electronic data sources were selected. Being cited by Kitchenhamet et al. [26] and Chen et al. [27] as relevant sources, the digital libraries were also selected because of the ease of access, the possibility of obtaining full text publications, and the fact that they are used for indexing journals and conference proceedings.

In order to allow a broader scope of the SLR, no limitations for the period of the publications were defined and only papers in English were selected for been considered the standard language of most international journal and conference proceedings. 

It should be added that Google Scholar was not included as a data source because of the high possibility of returning inaccurate results and the considerable overlap with ACM and IEEE electronic databases [27]. Table A1 (Appendix A) presents the selected studies retrieved after the execution of the research string, and Table A2 (Appendix A) the mapping regarding the selected digital libraries. 

### 3.3. Inclusion and Exclusion Criteria

In order to allow only relevant studies that met the objectives of the SLR to be returned after the execution of the search string, the inclusion and exclusion criteria presented in Table 3 were adopted.

### 3.4. Study Selection and Data Extraction

**Figure 3:**
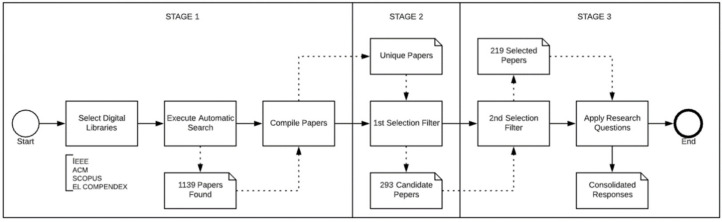
Publication’s selection process stages.

Regarding the publication’s selection process, three stages were performed. In the first stage, the electronic bases were selected, the research string was executed in each digital library (on 30 June 2021), and the returned publications were compiled resulting in 1139 papers. Figure 3 presents the steps of the performed study selection and data extraction.

In the second stage of the publication’s selection process, duplicate papers were discarded, and the first filter was performed, where title, keywords and abstract were read and inclusion and exclusion criteria were applied, resulting in 293 candidate publications.

In the third stage, a second filter was performed where, after all papers were downloaded, a full-text reading of each article was performed applying the inclusion and exclusion criteria, which resulted in 219 papers. Finally, the proposed research questions were applied in all studies where data extraction and answers recording were performed based on the terms presented in Table 4 (results and discussions are presented in Section 4). 

Table 4 presents the items adopted in the study in order to document the work, meet the research questions and evaluate the quality of the studies. The adopted quality criteria (Q1 to Q7) are described in Section 4.2 and the evidence levels, adopted in order to evaluate the maturity of the techniques described in the selected publications, are listed in Table 5. 

Based on the work presented by Chen et al. [28], Table 5 presents the classification items in which the selected publications were validated or evaluated in order to identify the level of evidence of the solutions described.

During the execution of the publication’s selection process, the selected studies were presented to another two research studies where, after selection agreement, the publications were classified and categorized (divergences cases in paper selection and/or classification were solved after discussions in order to ensure the inclusion of relevant papers in this study).

## 4. Results and Discussions

In this section, results and discussions regarding the synthesis and analysis of the data extracted from the selected studies to answer the research questions are presented (including demographic data information). 

### 4.1. Demographic Data

In order to provide an overview of the studies regarding publication venues, citation count, distribution by year of publication and countries, this section presents the demographic information on the selected studies. All included publications are listed in Table A1 (Appendix A).

#### Publication Venues and Citation Count

Information regarding publication venues and citations may be potentially useful for researchers interested in conducting research on a relevant topic, as well as partially show the impact of a study, the quality or the maturity of the proposed techniques. This is why it is also important to provide information on the distribution of the selected works on publication venues (as presented in Table A3—Appendix A), as well as an overview of the citation count.

Table A4 (Appendix A) presents an overview of the citation count of the selected publications sorted in descending order (information obtained from Google Scholar on 1 August 2021). 

From the descending ordered list in Table A4, it is possible to identify the publications which were most cited where, comparing the 10 most cited studies with the application domain categories as presented in Section 4.3.1, it is possible to identify that six publications (S5, S101, S102, S116, S125, and S132) adopted approaches focusing on pandemic planning application domain (CD4). Tracking, surveillance and Contact tracing (CD6) application domain contained three studies (S114, S208, and S212), and Healthcare and Clinical management (CD1) one study (S104).

Regarding adopted support mechanisms, analyzing the table mapping presented in Section 4.3.2, from the 10 most cited studies, seven publications (S5, S101, S102, S104, S116, S125, and S132) adopted data and mathematical application related solutions, two studies (S114 and S212) adopted internet of things and hardware, and three studies adopted Software/Systems/Apps/Programing languages as support mechanisms (some studies used more than one category of support mechanism). 

**Figure 4:**
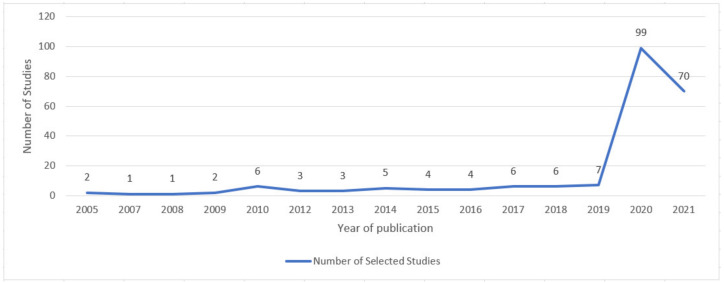
Number of selected papers published per year.

In Figure 4, the number of selected papers published per year is presented where, from 2020 onwards, a considerable increase in studies can be observed which, as mentioned before, is the period that the COVID-19 pandemic began. In other words, based on these data, it is possible to note that only after a global epidemic crisis emerged that the number of studies focused on the application of technology in primary ways of preventing the transmission of respiratory infectious diseases increased.

Table A5 (Appendix A) presents the distribution of the papers regarding the domain application categories (see Section 4.3.1) and the authors’ institution country. China, United States and India were the countries that presented the largest number of papers (48, 38 and 31, respectively), representing 53.42% of the publications selected in this study.

### 4.2. Study Quality Assessment

To perform the study quality assessment, the 219 studies were evaluated by the authors adopting the set of questions listed in Table 6, which were adopted and adjusted from the work presented by Chen et al. [28] and Dyba et al. [29]. Unlike the quality study proposed by the authors, the questions were not used to select the studies, but to validate the results.

During the analysis of the studies (see Section 3.4), each of the questions was answered according to a ratio scale (“Yes,” “No” and “Partially”), in order to obtain information about the credibility of the results. As mentioned by Kitchenhamet et al. [26] and Chen et al. [28], the result of quality assessment may also reveal potential limitations of current research and guide future field studies.

As presented in Table 6, the answers regarding the objectives and goals of the studies (Q1) were all positive and, regarding the context definition (Q2), more than 92% of the studies defined them clearly.

Information regarding the nature and type of the organization, adopted software, team experience and research design to achieve the objectives (Q3) was provided in almost 94% of the studies, and about 75% presented an adequate description of the data analysis (Q4) as well as presented a clear statement of the findings (Q5).

However, the greatest concern was about the examination of bias or influence in the study (Q6), where about 83% did not provide enough (or any) information, as well as in discussions about the study limitations (Q7), where 46.6% of the papers did not present discussions.

### 4.3. Question Analysis

In the following sub-sections, the analysis and discussions of the research questions will be presented.

#### 4.3.1. Available Approaches (RQ1) and Application Domains (RQ2) 

During the studies analysis and information extraction phase, the proposed approaches were identified (descriptions presented in Table A1), and the application domains were recognized and grouped into six main categories. Table 7 presents the proposed application domain categories (the number of studies for each category is presented in parentheses after the category name).

The categories descriptions are presented below:

- Healthcare and clinical management (CD1): Category that covers approaches that seek to adopt technological solutions focusing on healthcare, case investigation, medical supplies, among others like the ones that seek to diagnose infected individuals, monitor clinical status, predict clinical outcomes, and provide capacity for telemedicine services, virtual care, and hygiene surveillance.
- Infection testing/screening (CD2): A category that covers approaches focusing on screening/testing individuals for diseases, either assessing for signs of disease in an apparently asymptomatic population, for example, or adopting technology with medical procedures to confirm the diagnosis in individuals.
- Mask detection (CD3): Covers approaches that adopt information technology solutions aiming to detect people who are (or are not) using the protective mask.
- Pandemic Planning (CD4): Covers approaches that aim at the identification/obtainment of new information that can be used or contribute to the prevention and/or control of transmission of infectious diseases, including anticipation of behaviors, transmissions, new outbreaks of epidemics, among others.
- Quarantine/isolation/containment/social distancing (CD5): Category of approaches involving the application of technology in order to restrict the spread of infection through the contribution to social distancing, containment or isolation of indeciduous; for example, monitoring quarantine patients, restricting social contact using global positioning systems or mobile phone applications, among others.
- Tracking, surveillance, and Contact tracing (CD6): Include approaches that aim at the identification, tracking or tracing of individuals who might have come into contact with an infected person in order to tracks viral spread; for example, monitors the spread of infection across locations, or to prevent onward transmission by alerting those who came in contact with the positive case.

Of the selected studies, we can highlight that most of the works (about 61.19%) focused on pandemic planning (CD4) and healthcare and clinical management (CD1) related application domains, obtaining a percentage of about 34.25% for the CD4 category, and 26.94% for the CD1 category.

Regarding tracking, surveillance, and contact tracing (CD6)-related approaches, 31 studies (14.15%) were found and, with respect to Quarantine/isolation/containment/social distancing (CD5) application domain, 24 studies (10.96%) were identified.

Mask detection (CD3) and infection testing/screening (CD2)-related application domains were the ones with the fewest studies with only 7.31% and 6.39%, respectively.

**Figure 5:**
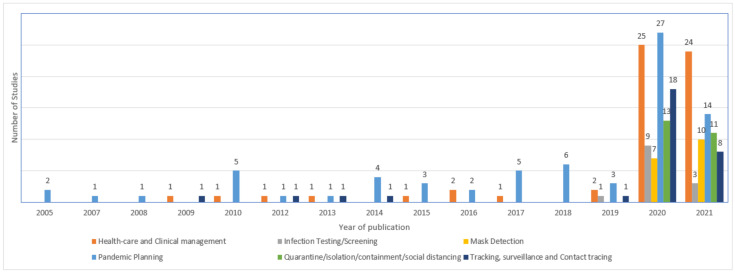
Distribution of studies by application domain and year of publication.

Figure 5 presents the quantitative distribution of studies by application domain category regarding the period of publication, where it is possible to observe that studies related to the categories CD3 and CD5 only appeared after 2020 and it was only in 2019 that studies related to the CD2 category emerged in publications.

It should be added that the studies were categorized regarding application domains that received greater prominence; although, in some cases, some fell into more than one category. Studies with more than one application domain were: S10 and S693 (CD3 and CD5); S53 and S79 (CD5 and CD6); S82 and S185 (CD2 and CD3); S109 (CD2 and CD5); and S121 (CD1 and CD3).

#### 4.3.2. Adopted Support Mechanisms (RQ3) 

After the identification of application domain categories of the selected studies, another important step would be the identification and categorization of the adopted/proposed support mechanisms. The mapping of the identified mechanisms according to the proposed categories (and sub-categories) is presented in Table 8 (the number of studies for each support mechanism is presented in parentheses).

Finishing the studies analysis and extraction of the support mechanisms information, four general categories (and nine sub-categories) were identified and adopted in order to group the selected studies. It should be added that it is common that some studies adopted more than one support mechanism, so they were included in more than one category.

Regarding studies that adopted algorithms, theories, mathematical/statistical models, a greater tendency was observed in the application of SEIR model, Gray Prediction Model, DSGE Algorithm, SLIR, SIS, SIR (24 studies), followed by Markov Model, Spatial Temporal Method, Graph Theory, NHPP, and Monte Carlo (19 studies). This grouping of studies was due to the fact that these studies used most of these mathematical models in their approaches or evaluations.

Regarding studies that applied artificial intelligence, deep learning, machine learning, big data and data mining, the greatest trend of the approaches applications was in neural network, feature enhancement module (FEM), spatial separable convolution, and SSD with a total of 41 studies.

Most of the studies that adopted mathematical models or machine learning focused on prediction for decision-making (or simulations) regarding present or future actions in pandemics, such as vaccination, social isolation, disease transmission and control, among others (see Table A1 for descriptions of the proposed approaches). In addition to assistance in increasing the capacity and accuracy of identification of infectious diseases cases and their expansion, artificial intelligence, machine learning, and big data also received a lot of attention from studies that focused on screening, contact tracing, and diagnosis of infectious diseases. 

Regarding support mechanisms related to software/systems/apps/programing languages (CS2) category (with 66 papers), studies were included that used existing market paid/free software or developed software/mobile apps as research contributions (programming languages and database management systems were also included).

With regard to studies that proposed Mobile, Desktop, WEB or Cloud applications or frameworks (see Table A1 for details of the approaches), for the case of Web and Cloud applications, there was a trend towards solutions focused on support for decision making, disease surveillance, and issuing alerts of critical areas with higher incidences of disease cases.

For Mobile applications, there was a greater tendence in contact tracing, social distancing, and body-symptoms detection/analysis. Regarding Desktop application, the proposed solutions focused on the containment of infectious disease outbreaks using geographical information with mathematical methods/models’ application.

Although many studies have proposed software or mobile apps as contributions in their approaches, there was a lack of detailed information regarding the adopted programming languages and database management systems (see Table 8). These situations usually occurred more in studies that also proposed the application of algorithms, theories, mathematical/statistical models and/or artificial intelligence, deep learning, machine learning, big data and data mining (CS2).

Regarding mobile applications, despite the lack of detail in the adopted programming languages or database management systems, most studies reported that the applications were developed with versions available for smartphones with Android and IoS (Apple), except for studies S72, S73, S92, S150, S151, and S194.

Regarding the adoption of internet of things and hardware (category CS3), most studies adopted sensors like environment (26 studies) and body (14 studies) sensors. In the case of environment sensors, there was a greater trend in the adoption for screening potential infectious diseases carriers from distance (e.g., temperature measurement/scanning), local position or movement measurement, automation of devices for hygiene (e.g., hand hygiene), and monitoring occupancy of places (e.g., monitoring elevator occupancy using a Passive Infrared (PIR) sensor).

Regarding studies that adopted wearable and/or mobile body sensors (14 studies), there was a greater tendence towards the verification of a Pearson’s heart rate, temperature, blood oxygen level and blood pressure. Video and photo cameras (11 studies) were used in devices such as smartphones, tablets, smart gates, cabins, doors, among other places, in order to collect video and/or image for purposes such as detecting use of masks, population monitoring, among others.

To enable communication between various IoT devices (e.g., mobile devices communicating among each other or with a centralized access point or server), devices with bluetooth/wifi/wireless technology (21 studies) or with RFID technology (9 studies) were adopted. 

Desktops, laptops and computer accessories such as memory cards, processors, and other boards (e.g., Raspberry pi, Arduino Uno, BeagleBoard-Xm, AT89S52 microcontroller, and others) have been adopted in 21 studies as accessories for the proposed approaches (see Table A1 for descriptions of the proposed approaches) or to be adopted with others support mechanisms, as well as printers and scan devices (3 studies). Manufacturer names and device versions were not considered for the mapping of the identified hardware.

Along with sensors and cameras, drones and/or unmanned aerial vehicles were adopted in 14 studies, with a greater tendency in population monitoring for purposes such as information collection of social distancing and contact tracing. 

Regarding surface disinfection in private or public spaces (including hand hygiene), automatic sprays and robots were adopted (see Table A1 for descriptions of the proposed approaches) and, with regard to studies that adopted Blockchain (7 studies), there was also a greater concern with the security in the sending and storage of the user data.

**Figure 6:**
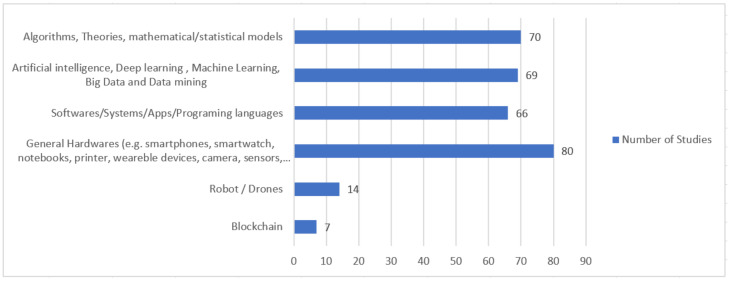
Quantitative distribution of the studies by support mechanisms.

In Figure 6, the quantitative distribution of the selected papers by sub-categories of support mechanisms is presented.

Of the selected studies, there was a greater tendency (139 studies) to adopt data and mathematical application related solutions (CS1), where most studies focused on the application of algorithms mathematical/statistical models (70 studies), and artificial intelligence, deep learning, machine learning, big data and data mining (69 studies).

The second category that showed greater adherence (94 studies) regarding the use/proposal of support mechanisms was related to the general use of internet of things and hardware (CS3) such as, for example, drones, robots, smartphones, smartwatch, wearable devices, camera, sensors, among others. 

#### 4.3.3. Available Evidence (RQ4) and Context Application (RQ5)

In order to obtain information regarding available evidence (RQ4) and context application (RQ5), data extraction was performed based on the items presented in Table 4, also aiming to investigate the maturity of the selected studies.

In Table 9, the distribution of the studies regarding the evidence levels (as described in Table 5) and context application (academic or industrial) is presented (the number of studies for each context/evidence level is presented in parentheses).

From the distribution presented in Table 9, only 13 studies did not present any evidence and, regarding demonstrations or examples, 36 studies presented application descriptions in the academic context.

Most studies carried out experiments both in academic contexts (65 studies) with fictitious data, and in industrial contexts (52 studies) with data obtained from real case scenarios. Expert observations such as textual, qualitative or opinion evaluations were provided in eight studies limited to the academic context.

Adding the studies carried out in laboratories (117 papers) with the studies that carried out both empirical investigation (46 papers) and strict analysis (22 papers), it can be seen that studies aimed at preventing the transmission of infectious diseases through the use of technology have, for the most part, some evidence with tests (total of 163 papers).

As the studies analyzed in this systematic review focus on the application of technologies in the primary ways of preventing the transmission of respiratory infectious diseases, it is also interesting to identify the context in relation to the diseases. Thus, Table 10 presents the distribution of studies regarding diseases in which the use of technology was proposed to contribute to their prevention (the number of studies for each disease category is presented in parentheses).

It should be added that the use of the term “Infectious diseases in general” was adopted to group studies that, despite using or not diseases as an example, have their approach generalized to infectious diseases in general, whose transmission can be reduced through primary forms of prevention, such as social distancing, mask usage, isolation and quarantine. Therefore, they were grouped separately from those studies whose approaches were proposed specifically for the diseases listed in Table 10.

As presented in Table 10, it is possible to identify a greater concentration of studies focused on the use of technology in the primary ways of preventing COVID-19 (139 studies), followed by infectious diseases in general (62 studies).

## 5. Research Implications and Limitations

As mentioned earlier, in order to obtain the greatest possible number of relevant studies in the systematic review, a comprehensive search was performed with automatic searches in digital libraries adopting terms related to the subject matter of the search. To contribute to the selection of relevant studies, inclusion and exclusion criteria were applied in two stages.

Regarding possible threats to the validity of this systematic review, we can mention the possibility of bias in the selection of publications and in data extraction where, due to the possibility of subjectivity in decision making, researchers’ bias can affect the results of the work.

To contribute to the reduction in bias in the selection of publications, the review protocol was initially developed by a researcher, and validated by two other researchers with extensive knowledge and experience in software engineering and in works related to systematic literature reviews. After completion of the review protocol, it was strictly followed.

The selection process of the studies was then conducted in three stages (see Section 3.4) to reduce the chances of exclusion of relevant studies, and to contribute to bias reduction. The study selection process was conducted by one researcher, and all included and excluded studies were examined by two other researchers (inclusion and exclusion motivations were recorded and disagreements were resolved through discussions).

The option of adopting only automatic searches in digital libraries was also chosen as a way to contribute to the reduction in bias in the search and selection of studies. The terms adopted in the searches were iteratively improved based on the evaluation searches and were carefully tested before the execution of the systematic review.

In order to reduce threats regarding data extraction inaccuracies, a data extraction form was created (as presented in Table 4) to enable a more consistent extraction and analysis of data to answer the proposed research questions of this systematic review.

To contribute to bias reduction, the data extraction procedure was performed by two researchers who executed the data extraction and verification of the selected studies performing a complete reading of the works and answering the research questions and the quality criteria according to Table 4. After recording and analyzing the extracted information, similar positions and conclusions were unified, and disagreements were resolved through discussions.

After obtaining the results, the third researcher validated them, acting as the final decision-maker for discussions when no agreement was made.

The quality assessment of the papers also contributed to the increase in accuracy and precision of data extraction process, giving more credibility to the fact that extracted data comes from reliable studies.

The major limitations of the studies are the limited number of selected sources (only four digital libraries), and the fact that only automatic searches were performed, where the use of keywords may not cover all studies that use technology in the primary forms of prevention of transmission of infectious diseases. 

This may happen because the searched terms may not be explicit in the title, keywords, or abstracts of the studies, as well as the string search, which may not contain the full set of terms required to obtain all the relevant works available in the digital libraries.

Another limitation that should be added is that, although the guidelines suggested by Kitchenhamet et al. [26] were adopted in this systematic review, instead of having the participation of a group of researchers in the data extraction, only three participated. Although the performance of analysis activity by a single researcher is common in many studies, it can also lead to questions regarding the description or classification of the collected data. To contribute to the reduction in bias in this scenario, three researchers participated in the study.

## 6. Conclusions

In the race against the spread of transmissible infectious diseases, there has been a growing interest in the use of technological solutions in the primary ways of preventing the transmission of these diseases. 

Due to the importance of the subject, mainly due to its economic and social impact to society, it is equally important to systematically identify, analyze and document what is being carried out and studied, discovering new directions, as well as having a better understanding of how technologies are being used in this scenario, its main objectives, adopted support mechanisms, level of evidence reported, gaps that need to be deepened in research, as well as to organize the knowledge to support the technological transition.

For this purpose, this work presented the design, execution, and results of a comprehensive systematic literature review of relevant studies on information technology applications in the primary ways of prevention of new infectious diseases transmission.

Based on the findings, it was possible to identify issues relevant to interested researchers and practitioners, as well as contributing to the availability of an evidence-based guide to select appropriate technologies, approaches, solutions, or support mechanisms based on the different needs or scenarios.

From the results presented in this literature review, it was possible to identify six application domain categories of the selected studies in which there was a greater trend in studies related to pandemic planning and, among the support mechanisms adopted, data and mathematical application related solutions received greater attention.

From the mapping of support mechanisms carried out, it was also possible to identify a trend towards the application of artificial intelligence, deep learning, and machine learning technologies in primary ways of preventing transmission of respiratory infectious diseases. Thus, a thorough analysis and comparison of these algorithms (e.g., analysis of the success rate of the algorithms) is proposed as future works.

Regarding quality assessment analysis, most of the studies did not provide enough (or any) information about the examination of bias or influence in the study, as well as in discussions about the study limitations. Regarding available evidence, most of the studies presented some evidence (with tests).

From the findings, a greater tendency of studies focused on the use of technology in the primary ways of preventing COVID-19 was identified, followed by infectious diseases in general.

While it cannot be said that the study is exhaustive, it is believed to be a useful resource for interested researchers and practitioners regarding the use of technological solutions in the primary ways of preventing the transmission of infectious diseases.

For future works, it is recommended to expand the scope of this study with manual searches, including with searches in the references of the selected studies (through snowball techniques).

## Figures and Tables

**Table 1:** Proposed research questions (RQ).

Research Question	Motivation
RQ1: Which approaches have been adopted or suggested in the primary ways of preventing respiratory infectious diseases using information technology solutions?	The purpose is to identify infectious diseases transmission prevention approaches that are proposed or applied with the use of information technology
RQ2: What are the application domains of the identified approaches?	Identify the application domains of the approaches. With this information, researchers and practitioners can identify the application domains that have gained the most interest in the primary forms of prevention of infectious diseases
RQ3: Which support mechanisms are proposed or used?	What technologies, systems, techniques, among other mechanisms have been proposed or adopted to support or achieve the objectives of the approaches? This knowledge can help practitioners and/or researchers in the identification of trends in the use of technological solutions as support mechanisms.
RQ4: How much evidence to support the adoption of the approaches is available?	Obtain knowledge about the maturity of the identified approaches, in order to assist researchers and practitioners in further adoption or evaluation of existing approaches in this systematic review, maturity was measured based on levels of evidence (see Section 3.4).
RQ5: Which contexts are addressed?	Identify the contexts (academy or industry) in which the studies were applied, validated or evaluated. For industrial context, real case data or evaluation in real case scenarios is considered. If validations were described in both contexts, the industrial context is considered for the purpose of evaluating the works.

**Table 2:** Electronic databases adopted in the automatic searches.

Electronic Database	Search Terms Matched With	Web Address	Publications Found
IEEE Xplore Digital Library	Paper title, keywords, abstract	http://ieeexplore.ieee.org	117
ACM Digital Library	Paper title, keywords, abstract	http://dl.acm.org	94
El Compendex	Paper title, keywords, abstract	http://engineeringvillage.com	487
Elsevier Scopus	Paper title, keywords, abstract	http://www.scopus.com	441

**Table 3:** Adopted inclusion and exclusion criteria.

**Inclusion Criteria**
I1: Infectious diseases transmission/spread prevention-related works/approaches addressing the use of information technology solutions.
**Exclusion Criteria**
E1: Duplicate publications (including different references)
E2: Standards, models, industry standards
E3: Editorials, reports, position papers, keynotes, reviews, perspectives, surveys, summaries tutorials, books, courses or workshops, panel discussions
E4: Non-scientific publications
E5: Publications not related to infectious diseases transmission/spread prevention in humans
E6: Publications that do not cover “respiratory infectious diseases”, or whose approaches could not be applied in the primary ways of preventing transmission of these diseases
E7: Publications that do not have sufficient information to solidly answer the research questions
E8: Publications that do not meet the inclusion criteria

**Table 4:** Summary of items extracted from each study including research questions and quality criteria.

Objective	Item	Objective	Item
General Data	Title	RQ5	Context
	Author(s)	Q1	Objective of the Study
	Publication Year	Q2	Description of the Context
	Venue	Q3	Description of the Research Project
	Paper Summary	Q4	Analysis of the Data
RQ1	Approach	Q5	Conclusions Presentation
RQ2	Application Domain	Q6	Critical Analysis Description
RQ3	Adopted Support Mechanisms	Q7	Description of Limitations and Bias
RQ4	Level of Evidence		

**Table 5:** Levels of Evidence.

Level	Classification	Description
0	No evidence	No evidence was presented regarding evaluation or validation
1	Example or demonstration	Application description is provided with an example to aid its description
2	Specialists Notes	Qualitative or textual assessments are provided. Example: advantages and disadvantages contrasts/comparation
3	Experiment in laboratory	Results are reached from simulations with artificial data in real experiments. Evidence collection is performed formally or informally.
4	Empirical Investigation	Real context investigation of the behavior of the proposed approach
5	Strict analysis	Evaluation/validation of the study is performed using a formal methodology. Example: questions and variables definition for analysis after the application of the approach

**Table 6:** Quality assessment questions.

ID	Quality Assessment Question	Yes	Partially	No
Q1	Are the study objectives and goals clearly specified?	218 (99.5%)	1 (0.5%)	0 (0.0%)
Q2	Is the study context clearly defined?	113 (51.6%)	89 (40.6%)	17 (7.8%)
Q3	Does the research design support the objectives/goals of the study?	135 (61.6%)	71 (32.4%)	13 (5.9%)
Q4	Does the study have an adequate description of the analysis of the data?	96 (43.8%)	67 (30.6%)	56 (25.6%)
Q5	Does the study present a clear statement of the findings and provide enough data to support them?	79 (36.1%)	81 (37.0%)	59 (26.9%)
Q6	Do researchers critically examine potential bias and/or influence in the study?	3 (1.4%)	33 (15.1%)	183 (83.6%)
Q7	Study limitations are discussed explicitly?	51 (23.3%)	66 (30.1%)	102 (46.6%)

**Table 7:** Application Domain Categories.

Category	Studies
CD1: Healthcare and Clinical management (59)	S4, S11, S14, S21, S24, S25, S27, S31, S37, S38, S39, S40, S56, S57, S58, S59, S60, S61, S64, S70, S77, S80, S88, S89, S92, S93, S95, S103, S104, S112, S123, S126, S127, S133, S137, S139, S140, S141, S150, S151, S157, S158, S160, S168, S171, S181, S182, S183, S187, S191, S192, S197, S202, S203, S205, S206, S207, S218, S219
CD2: Infection Testing/Screening (14)	S26, S35, S65, S82, S118, S122, S128, S148, S159, S163, S177, S185, S193, S198
CD3: Mask Detection (16)	S6, S8, S47, S63, S74, S75, S76, S106, S107, S108, S121, S142, S143, S144, S152, S178
CD4: Pandemic Planning (75)	S1, S2, S3, S5, S7, S15, S17, S23, S28, S29, S32, S36, S41, S43, S44, S45, S46, S48, S51, S53, S54, S55, S62, S66, S68, S71, S78, S81, S83, S85, S91, S96, S97, S98, S99, S100, S101, S102, S105, S111, S113, S116, S117, S120, S124, S125, S129, S130, S132, S138, S145, S146, S147, S153, S155, S156, S161, S164, S165, S169, S170, S173, S180, S186, S188, S189, S190, S199, S200, S201, S204, S209, S211, S215, S216
CD5: Quarantine/isolation/containment/social distancing (24)	S10, S16, S19, S22, S30, S50, S52, S69, S79, S84, S90, S109, S110, S119, S131, S135, S167, S172, S174, S179, S184, S194, S195, S213
CD6: Tracking, surveillance, and Contact tracing (31)	S9, S12, S13, S18, S20, S33, S34, S42, S49, S67, S72, S73, S86, S87, S94, S114, S115, S134, S136, S149, S154, S162, S166, S175, S176, S196, S208, S210, S212, S214, S217

**Table 8:** Support Mechanisms.

Category	Sub-Category	Support Mechanism	Studies
CS1: Data and Mathematical Application Related Solutions	Algorithms, Theories, Mathematical/Statistical Models	Bootstrap Method (1)	S170
		Dijkstra Algorithm (1)	S16
		Discrete Fourier Transform (DFT) model (1)	S65
		General Algorithms, mathematical models/equations (12)	S9, S34, S49, S52, S69, S94, S102, S130, S156, S162, S210, S214
		K-nearest Neighbor Algorithm, Nearest-neighbour distance (2)	S13, S167
		Markov Model, Spatial Temporal Method, Graph Theory, NHPP, Monte Carlo (19)	S17, S18, S23, S34, S36, S44, S51, S53, S62, S85, S99, S101, S111, S116, S129, S149, S154, S215, S216
		Multi-agent (Model/simulation), Equation-based model (13)	S2, S3, S4, S5, S7, S91, S96, S99, S100, S125, S132, S160, S211
		Multiple Signal Classification (MUSIC) Algorithm (1)	S128
		Optimal Control Theory (1)	S161
		Regression models, Short-term Prediction, RMSE, MAE (5)	S15, S68, S128, S147, S169
		SEIR model, Grey Prediction Model, DSGE Algorithm, SLIR, SIS, SIR (24)	S21, S32, S55, S66, S91, S96, S97, S98, S116, S117, S129, S130, S145, S146, S153, S155, S161, S189, S190, S199, S200, S201, S209, S215
		Self-Propelled Entity Dynamics (SPED) model, LDS—Low Discrepancy Sequence (1)	S164
	Artificial intelligence, Deep learning, Machine Learning, Big Data and Data mining	Big Data (5)	S1, S42, S81, S123, S147
		Decision Tree, Regression Tree, CART (5)	S40, S41, S46, S123, S180
		DBSCAN—Density-Based Spatial Clustering of Applications with Noise (1)	S172
		Fuzzy Logic (3)	S125, S171, S192
		Heterogeneous Diffusion Network (1)	S154
		K-means (5)	S33, S44, S180, S214, S217
		LLA—Lexical Link Analysis (1)	S138
		Logistic Regression (10)	S27, S45, S46, S55, S104, S149, S154, S169, S170, S177
		Maximum Entropy Model (1)	S105
		Naive Bayes (2)	S27, S41
		NLP—Natural Language Processing (3)	S103, S137, S158
		Neural network (CNN, MTCNN, MobileNet, others), Feature Enhancement Module (FEM), Spatial Separable Convolution, SSD (41)	S6, S8, S10, S20, S40, S41, S45, S47, S48, S55, S57, S58, S59, S60, S61, S63, S64, S65, S70, S74, S75, S76, S88, S90, S106, S107, S108, S109, S112, S120, S121, S142, S143, S144, S152, S178, S182, S184, S185, S201, S206
		Random Forest, iForest (5)	S27, S33, S40, S41, S46
		Support Vector Machine (8)	S3, S27, S40, S41, S45, S46, S61, S104
		Vector Space Model (2)	S123, S141
CS2: Software/Systems/Apps/Programing languages	Market Software/Platform (Proprietary or Free/Open Source)	Android Studio (1)	S71, S135
		AnyLogic, Django Framework (1)	S2
		ArcGIS (3)	S28, S62, S208
		Autodesk Revit/Meshmixer, Rhino3D, AutoCAD, Grasshopper (4)	S83, S110, S188, S199
		AWS—Amazon Web Services (e.g., software, and load Balancer, elastic container, lambda, Greengrass, others) (3)	S12, S62, S137
		Bootstrap, Adobe Photoshop (1)	S204
		Business Model Canvas (BMC), Service Blueprint (1)	S124
		Ethereum (1)	S67
		Google Cloud Platform (2)	S62, S68
		Hadoop (1)	S42
		Hyperledger Fabric (1)	S117
		IOTA Tangle Platform (1)	S71
		Kibana (Elasticsearch) (1)	S62
		MATLAB (1)	S113
		Microsoft Azure Cloud (2)	S70, S78
		NetLogo (1)	S4
		NLTK—Natural Language Toolkit (1)	S158
		Node.js (2)	S62, S137
		Node-RED and Grafana (1)	S136
		OpenCV (2)	S109, S151
		Ultimaker Cura (1)	S110
		Unity Platform (e.g., WebGL, 3D) (3)	S83, S131, S179
		WeChat, WhatsApp, WhatsApp Bot (4)	S93, S109, S111, S117
		Wireshark Dumpcap (1)	S195
		Zoom Platform (1)	S207
	Mobile, Desktop, WEB or Cloud Application/Framework proposed as study contributions	Cloud Application (3)	S12, S31, S35
		Desktop Application (4)	S51, S83, S96, S113
		Mobile Application (24)	S12, S20, S33, S63, S70, S72, S73, S84, S92, S111, S115, S119, S123, S135, S150, S151, S165, S166, S175, S192, S194, S203, S204, S214
		Web Application/Framework (21)	S2, S14, S28, S29, S37, S41, S42, S62, S67, S68, S78, S82, S93, S123, S131, S158, S165, S166, S176, S204, S214
	Programming Languages	C#, C++ (2)	S83, S96
		Java (J2EE, J2ME, JNI, Hibernate) (5)	S14, S28, S37, S73, S92
		JavaScript Libraries/ API (e.g., jQuery, ReactJS, AJAX, Google Web Toolkits, Google Maps) (9)	S2, S67, S28, S29, S72, S151, S166, S196, S204
		PHP (2)	S166, S204
		Python (6)	S2, S83, S96, S158, S199, S216
		Visual Basic (1)	S51
	Data Base Management System	Firebase (4)	S71, S73, S92, S194
		Influx DB (1)	S136
		MongoDB (2)	S2, S42
		MYSQL (3)	S78, S123, S204
		MS Access (1)	S51
		Oracle (1)	S29
		PostgreSQL (2)	S37, S208
		Neo4j (1)	S12
		SQLite (1)	S83
CS3: Internet of Things and Hardware	*^1^	Wearable devices (e.g.,smartwatches, smartphones, smartbelt, and others) (17)	S19, S27, S40, S54, S56, S69, S70, S79, S111, S115, S139, S157, S163, S175, S181, S195, S213
	Sensors (mobile or fixed), Cameras, RFID (Radio Frequency Identification)	Cameras—photo and video (Fixed and mobile) (11)	S30, S74, S82, S106, S109, S118, S121, S122, S128, S193, S194
		Environment Sensors (e.g., Passive Infrared (PIR) Sensor, and others) (26)	S11, S16, S21, S30, S38, S47, S50, S54, S74, S77, S82, S89, S109, S114, S121, S122, S126, S127, S135, S136, S172, S177, S185, S191, S192, S193
		RFID (Radio Frequency Identification) devices (9)	S13, S27, S30, S35, S50, S182, S185, S192, S196
		Wearable and/or mobile body sensors (e.g., temperature, cough, oxygen, pressure, heart rate measurement) (14)	S21, S25, S26, S27, S31, S35, S44, S69, S86, S87, S118, S157, S159, S181
	Others (e.g., Printers, Spray, Chips, GPS/GSM/Bluetooth devices, WIFI routers, UV tech, WBAN, and others)	Bluetooth/WIFI/GPS/Wireless devices (e.g., module, routers, access point, receivers, SMS gateways, GPS chips, and others) (21)	S9, S18, S26, S31, S44, S50, S84, S86, S123, S124, S134, S136, S142, S148, S172, S175, S194, S196, S207, S212, S219
		Desktops, Laptops, and computer accessories (e.g., memory cards, processors, and other boards) (21)	S25, S31, S39, S47, S74, S86, S109, S114, S121, S123, S134, S136, S137, S139, S185, S191, S192, S197, S198, S207, S219
		Printer and scan devices (3)	S110, S174, S218
		Spray/Dispenser devices (6)	S11, S82, S168, S191, S205, S219
		UV technology (e.g., UVC, UV Chip, UV Led, UV Light, UV ray) (7)	S11, S24, S38, S95, S127, S133, S148
	Robot/Drones	Robot/Drones/Unmanned Aerial Vehicles (UAV) (14)	S18, S22, S43, S80, S90, S127, S140, S167, S173, S183, S187, S198, S205, S218
CS4: Blockchain	*^1^	Blockchain (7)	S43, S67, S71, S117, S162, S173, S186

*^1^ Fields with “*” have no value.

**Table 9:** Study distribution regarding evidence level and context.

Evidence Level	Context	
	Academic (121)	Industrial (98)
0: No evidence (13)	S3, S11, S20, S25, S53, S56, S70, S124, S126, S141, S150, S177, S181,	*^1^
1: Example or demonstration (36)	S1, S5, S9, S12, S26, S30, S31, S33, S36, S50, S67, S69, S79, S81, S82, S84, S87, S101, S107, S127, S133, S134, S140, S146, S163, S165, S166, S175, S179, S185, S191, S193, S197, S208, S213, S214,	*^1^
2: Specialists Notes (7)	S14, S47, S71, S72, S86, S174, S194,	*^1^
3: Experiment in laboratory (117)	S2, S7, S8, S10, S13, S16, S18, S34, S40, S43, S44, S49, S51, S52, S54, S59, S62, S64, S66, S75, S80, S89, S90, S91, S94, S96, S98, S99, S100, S102, S103, S104, S105, S109, S112, S115, S119, S121, S122, S129, S132, S142, S145, S151, S153, S156, S158, S159, S161, S162, S164, S171, S172, S173, S180, S182, S186, S190, S200, S206, S209, S210, S211, S215, S219	S4, S6, S17, S23, S37, S38, S39, S41, S60, S61, S65, S68, S74, S76, S77, S92, S95, S106, S108, S110, S111, S113, S116, S117, S118, S120, S123, S125, S128, S136, S138, S143, S147, S148, S152, S155, S160, S167, S170, S183, S184, S187, S188, S189, S195, S196, S198, S199, S202, S204, S216, S218
4: Empirical Investigation (24)	*^1^	S19, S22, S27, S28, S29, S32, S46, S55, S73, S83, S85, S88, S93, S130, S131, S135, S139, S154, S169, S176, S203, S205, S207, S212,
5: Strict analysis (22)	*^1^	S15, S21, S24, S35, S42, S45, S48, S57, S58, S63, S78, S97, S114, S137, S144, S149, S157, S168, S178, S192, S201, S217

*^1^ Fields with “*” have no value.

**Table 10:** Papers distribution according to the studied diseases.

Diseases	Studies
Infectious diseases in general (using or not some disease as examples) (62)	S1, S3, S4, S5, S13, S21, S23, S28, S29, S36, S37, S42, S49, S53, S76, S85, S96, S101, S103, S105, S111, S113, S117, S118, S122, S123, S124, S126, S128, S129, S130, S132, S133, S146, S147, S149, S150, S151, S154, S156, S159, S160, S161, S162, S164, S165, S168, S172, S173, S177, S181, S182, S186, S187, S196, S197, S204, S208, S211, S213, S215, S219
COVID-19 (139)	S6, S8, S9, S10, S11, S12, S14, S15, S16, S18, S19, S20, S22, S24, S25, S26, S27, S30, S31, S32, S33, S34, S35, S38, S39, S40, S41, S43, S44, S45, S46, S47, S50, S52, S54, S55, S56, S57, S58, S59, S60, S61, S62, S63, S64, S65, S66, S67, S68, S69, S70, S71, S72, S73, S74, S75, S77, S78, S79, S80, S81, S82, S83, S84, S86, 87, S88, S89, S90, S92, S93, S94, S97, S98, S104, S106, S107, S108, S109, S110, S112, S119, S120, S121, S127, S131, S134, S135, S136, S137, S138, S139, S140, S141, S142, S143, S144, S145, S148, S152, S153, S157, S158, S163, S166, S167, S169, S170, S171, S174, S175, S176, S178, S179, S180, S183, S184, S185, S188, S189, S191, S192, S193, S194, S195, S198, S199, S200, S202, S203, S205, S206, S207, S209, S210, S214, 216, S217, S218
Influenza (H1N1, H5N1, and others) (17)	S2, S7, S17, S48, S51, S91, S99, S100, S102, S114, S115, S116, S125, S155, S190, S201, S212
Klebsiella pneumoniae (1)	S195

## Data Availability

Not applicable.

## Appendix A

See Table A1, Table A2, Table A3, Table A4 and Table A5.

**Table A1:** Selected Publications.

ID	Reference	Title	Approach	Venue Acronym	Year
S1	[30]	A big data architecture to a multiple purpose in healthcare surveillance: the Brazilian syphilis case	Big data architecture to a multiple purpose in healthcare surveillance	EATIS	2020
S2	[31]	A computational paradigm for the simulation of complex epidemic diseases	Paradigm for modeling and simulation of complex system using agent-based model	SoICT	2016
S3	[32]	A development of an efficient information collecting and retrieval system using an Agent Technology for infectious disease	Information retrieval system with agent technology for the field of infection disease	Healthcom	2005
S4	[33]	A dynamic patient network model of hospital-acquired infections	Agent-based model to simulate the effect of network density and patient sharing on transmission	WSC	2010
S5	[34]	A hybrid epidemic model: combining the advantages of agent-based and equation-based approaches	Model and initial examples of a threshold hybrid model that switches between agent-based and equation-based descriptions	WSC	2017
S6	[35]	A Mask Detection Method for Shoppers Under the Threat of COVID-19 Coronavirus	Single-shot detector (SSD) based method for face masks detection in the supermarket	CVIDL	2020
S7	[36]	A multi-agent model for adaptive vaccination during infectious disease outbreaks	Age-structured multi-agent model to simulate an epidemic spread	ICCTIDE	2016
S8	[37]	A Network for Detecting Facial Features During the COVID-19 Epidemic	Facial feature detection algorithm based on Mtcnn + Mobilenet + GDBT	CCEAI	2021
S9	[38]	A note on blind contact tracing at scale with applications to the COVID-19 pandemic	Cryptographic approach to contact tracing based on secure two-party computation	ARES	2020
S10	[39]	A Novel COVID Prevention Method Using Deep Learning	PC vision and MobileNet V2 architecture adoption in social distancing and mask detection	ICPECTS	2020
S11	[40]	A Novel Method of Mass Disinfection for the Prevention of COVID-19	Technology solution that adopts Ultraviolet–C light exposure along with disinfectant spray	IJCRR	2021
S12	[41]	A Privacy-Preserving Mobile and Fog Computing Framework to Trace and Prevent COVID-19 Community Transmission	E-government Privacy-Preserving Mobile, and Fog computing framework to trace infected people	IEEE J Biomed Health Inform	2020
S13	[42]	A RFID-Based Infection Prevention and Control Mechanism in Aged Care Living Residences	Visual infection control positioning system for aged care facilities	Mob. Netw. Appl.	2021
S14	[43]	A Service-oriented Framework for Developing Personalized Patient Care Plans for COVID-19	Service oriented framework that allows for dynamic composition and management of personalized patient care	DG.O	2021
S15	[44]	A Short-Term Prediction Model at the Early Stage of the COVID-19 Pandemic Based on Multisource Urban Data	Short-term prediction model for COVID-19 cases	TCSS	2021
S16	[45]	A Social IoT-Driven Pedestrian Routing Approach During Epidemic Time	Pedestrians safely navigation framework with Social IoT adoption	GCAIoT	2020
S17	[46]	A Spatial–Temporal Method to Detect Global Influenza Epidemics Using Heterogeneous Data Collected from the Internet	Spatial–temporal method that incorporates heterogeneous data collected from the Internet to detect influenza epidemics	TCBB	2018
S18	[47]	A Spatio-GraphNet Model for Real-time Contact Tracing of COVID-19 Infection in Resource Limited Settings	Spatio-GraphNet model for real-time contact tracing of COVID-19 infection	ICMHI	2020
S19	[48]	A wearable magnetic field based proximity sensing system for monitoring COVID-19 social distancing	Wearable, oscillating magnetic field-based proximity sensing system to monitor social distancing	ISWC	2020
S20	[49]	Active Surveillance for COVID-19 Through Artificial Intelligence Using Real-Time Speech-Recognition Mobile Application	Model of active surveillance for COVID-19 through artificial intelligence	ICCE-Taiwan	2020
S21	[50]	An Alternative Body Temperature Measurement Solution: Combination of a Highly Accurate Monitoring System and a Visualized Public Health Cloud Platform	Body temperature monitoring system with a thermography based on IoT architecture	IEEE Internet Things J	2021
S22	[51]	An Autonomous Delivery Robot to Prevent the Spread of Coronavirus in Product Delivery System	Prototype robot to reduce the risk of infectious disease transmission in the product delivery system	UEMCON	2020
S23	[52]	An Efficient Immunization Strategy Using Overlapping Nodes and Its Neighborhoods	Address the effect of nodes in the neighborhood of the overlapping nodes on epidemics spreading	WWW Companion	2018
S24	[53]	An emerging innovative uv disinfection technology (Part ii): Virucide activity on SARS-CoV-2	UV chip technology adoption in viral charge reduction	IJERPH	2021
S25	[54]	An enhanced, contactless, IoT based operation of power appliances for COVID-19 isolation zone	IoT adoption for power appliances devices without physical contact	JGE	2020
S26	[55]	An integrated biosensor system with mobile health and wastewater-based epidemiology (iBMW) for COVID-19 pandemic	Integrated biosensor system with mobile health and wastewater-based epidemiology	JBSBE	2020
S27	[56]	An Intelligent and Energy-Efficient Wireless Body Area Network to Control Coronavirus Outbreak	Novel coronavirus-body area network model based on IoT technology monitoring system for the detection of coronavirus infection	Arab J Sci Eng.	2021
S28	[57]	An Interactive Web-based Decision Support System for Mass Dispensing, Emergency Preparedness, and Biosurveillance	Interactive web-based real-time decision support suite that allow public health emergency preparedness coordinators	DH	2017
S29	[58]	An Interactive, Web-Based High Performance Modeling Environment for Computational Epidemiology	An integrated interactive modeling environment to support public health epidemiology	TMIS	2014
S30	[59]	An Internet of Things Paradigm: Pandemic Management (incl. COVID-19)	IoT paradigm to strengthen the old-style public health measures	ICAIS	2021
S31	[60]	An IoT-Cloud Based Health Monitoring Wearable Device for COVID Patients	Use of mobile apps and remote health monitoring system that uses IoT for health diagnosis	ICACCS	2021
S32	[61]	Analysis of 2019-nCoV epidemic situation based on modified SEIR model and DSGE algorithm	Prediction model based on the SEIR infectious disease transmission model to help prediction of the pandemic outbreak	ISCTT	2020
S33	[62]	Analysis of COVID-19 Tracking Tool in India: Case Study of Aarogya Setu Mobile Application	Exploration of a mobile technology for tracking or contact tracing	DGOV	2020
S34	[63]	Application of semantic location awareness computing based on data mining in COVID-19 prevention and control system	Spatiotemporal trajectory data mining method application in epidemic prevention and monitoring	J. Intell. Fuzzy Syst.	2020
S35	[64]	Apply IOT technology to practice a pandemic prevention body temperature measurement system: A case study of response measures for COVID-19	Intelligent pandemic prevention Temperature Measurement System and Pandemic Prevention situation Analysis System	IJDSN	2021
S36	[65]	Approaching multi-dimensional cube for visualization-based epidemic warning system—dengue fever	Visualization-based warning system to control the development of epidemic	ICUIMC	2014
S37	[66]	Assisted Telemedicine Model for Rural Healthcare Ecosystem	Assisted Telemedicine model and app for Rural Healthcare Ecosystem	WebSci	2021
S38	[67]	AT89S52-Microcontroller Based Elevator with UV-C disinfection to prevent the transmission of COVID-19	System for the automatic disinfection of elevator cabins using UV-C light	ICPS	2020
S39	[68]	Automated AMBU Ventilator with Negative Pressure Headbox and Transporting Capsule for COVID-19 Patient Transfer	Automated AMBU ventilator	Front. Robot. AI	2021
S40	[69]	Avoid Touching Your Face: A Hand-to-face 3D Motion Dataset (COVID-away) and Trained Models for Smartwatches	COVID-away dataset and trained models	IoT Companion	2020
S41	[70]	Be Aware of the Hot Zone: A Warning System of Hazard Area Prediction to Intervene Novel Coronavirus COVID-19 Outbreak	Warning system to predict hazard areas in order to intervene the novel coronavirus COVID-19 epidemic transmission	SIGIR	2020
S42	[71]	Big Data Analytics Framework for Childhood Infectious Disease Surveillance and Response System using Modified MapReduce Algorithm A Case Study of Tanzania	Framework for Childhood Infectious Disease Surveillance and Response System	IJACSA	2021
S43	[72]	Blockchain-enabled secure communication for drone delivery: a case study in COVID-like scenarios	Blockchain-enabled secure communication framework for delivering the goods in COVID-19 like scenarios	MobiCom	2020
S44	[73]	Bridging Predictive Analytics and Mobile Crowdsensing for Future Risk Maps of Communities Against COVID-19	MCS-driven community risk modeling solution against COVID-19 pandemic	MobiWac	2020
S45	[74]	Can artificial intelligence enable the government to respond more effectively to major public health emergencies? Taking the prevention and control of COVID-19 in China as an example	3S technology adoption to propose the establishment of an emergency command platform for public health emergencies	Socio-Econ. Plan. Sci.	2021
S46	[75]	Characterizing the spread of COVID-19 from human mobility patterns and SocioDemographic indicators	Adoption of human mobility patterns and SocioDemographic indicators to examine the overall spread of COVID-19 at local spatial scales	SIGSPATIAL	2020
S47	[76]	CNN Based COVID-19 Prevention System	Artificial intelligent IoT system with temperature monitoring and mask detection	ICAIS	2021
S48	[77]	Cola-GNN: Cross-location Attention based Graph Neural Networks for Long-term ILI Prediction	Cross-location attention-based graph neural network for learning time series embeddings in long-term influenza-like illness predictions	CIKM	2020
S49	[78]	Contact tracing in healthcare digital ecosystems for infectious disease control and quarantine management	Novel approach for contact tracing to find the clusters of cases and the infection tree for SARS automatically	DEST	2009
S50	[79]	Contactless and context-aware decision making for automated building access systems	Context-aware building access system to improve infection control measures	CAADRIA	2021
S51	[80]	Containing acute disease outbreak	Development of a Geographical Information System for the containment of acute infectious diseases	Methods Inf. Med.	2005
S52	[81]	Controlling the Outbreak of COVID-19: A Noncooperative Game Perspective	noncooperative game to incentive social distancing to prevent the spread of COVID-19	IEEE Access	2020
S53	[82]	Controlling the Spreads of Infectious Disease and Scare via Utilizing Location and Social Networking Information	Location and social networking information adoption to control the spread of infectious disease and the scare	Mobidata	2015
S54	[83]	Convergence model of AI and IoT for virus disease control system	Virus disease control system using an IoT/AI convergence model to proactively detect and warn of risk factors in response to infectious diseases	Pers. Ubiquitous Comput.	2021
S55	[84]	Coronavirus Epidemic (COVID-19) Prediction and Trend Analysis Based on Time Series	Machine Learning application for epidemic development prediction	AIID	2021
S56	[85]	Cost-Effective Device for Autonomous Monitoring of the Vitals for COVID-19 Asymptomatic Patients in Home Isolation Treatment	Healthcare system for body vitals infection analysis	CoCoNet	2021
S57	[86]	COVID Detection from Chest X-rays with DeepLearning: CheXNet	Model for COVID prediction from chest X-rays using CheXNet	ICCCS	2020
S58	[87]	COVID-19 Chest CT Image Segmentation Network by Multi-Scale Fusion and Enhancement Operations	Deep convolutional neural network tailored for segmenting the chest CT images with COVID-19 infections	IEEE Trans. Big Data	2021
S59	[88]	COVID-19 Chest Radiography Images Analysis Based on Integration of Image Preprocess, Guided Grad-CAM, Machine Learning and Risk Management	Methodology for chest radiography images application, as one of workable alarm and analysis systems to support clinicians against COVID-19 outbreak threat	ICMHI	2020
S60	[89]	COVID-19 CT Image Recognition Based on Multi-stage Transfer Learning	Deep learning adoption to automatically distinguish subjects’ lung CT images to assist in the diagnosis of COVID-19	ICASIT	2020
S61	[90]	COVID-19 Detection Based on Image Regrouping and Resnet-SVM Using Chest X-Ray Images	Combination of image regrouping and ResNet-SVM to detect COVID-19 in chest X-ray images	EMBS	2021
S62	[91]	COVID-19 Joint Pandemic Modeling and Analysis Platform	All-encompassing operational platform for non-pharmaceutical interventions to the COVID-19 pandemic	SIGSPATIAL	2020
S63	[92]	COVID-19 Pandemic Prevention Mobile Application for on Campus Classroom	Mobile application for COVID-19 Pandemic Prevention	ICCCS	2021
S64	[93]	COVID-19 Pneumonia Detection in Chest X-ray Images Using Transfer Learning of Convolutional Neural Networks	Adopt CNN Model to diagnose the chest X-ray images of patients who have pneumonia by COVID-19	DSIT	2020
S65	[94]	COVID-19 Recognition Based on Patient’s Coughing and Breathing Patterns Analysis: Deep Learning Approach	Deep LSTM technique to diagnose and detect COVID-19 infection from cough, breath, and sneeze signals	FRUCT	2021
S66	[95]	COVID-19 Spread Prediction Model	COVID-19 Spread Prediction Model	CONFCDS	2021
S67	[96]	COVID-19: A Novel Framework to Globally Track Coronavirus Infected Patients using Blockchain	Novel blockchain-based framework that integrates intercountry for COVID-19 to track infected or tested patients globally	ICCI	2020
S68	[97]	COVID-19: Update, Forecast and Assistant—An Interactive Web Portal to Provide Real-Time Information and Forecast COVID-19 Cases in Bangladesh	Informative and prediction-based web portal	ICICT4SD	2021
S69	[98]	COVID-19′s Telemedicine Platform	Telehealth solution to support social distance control, effective contact detection, continuous monitoring of people’s health status	ICISA	2021
S70	[99]	CovidSense: A Smartphone-based Initiative for Fighting COVID-19 Spreading	Smartphone-based app to provide a reliable COVID-19 risk index	DSAI	2020
S71	[100]	CoviReader: Using IOTA and QR Code Technology to Control Epidemic Diseases across the US	Decentralized healthcare management system that shares user’s data anonymously	CCWC	2021
S72	[101]	COVTrac: COVID-19 Tracker and Social Distancing App	COVID-19 tracker and social distancing app	ICACIE	2020
S73	[102]	COWAR: An Android Based Mobile Application to Help Citizens and COVID-19 Warriors	Mobile application to track the spread of the COVID-19	CICT	2020
S74	[103]	Deep learning based mask detection in smart home entries during the epidemic process	Design of deep learning-based mask detection in smart home system	SCA	2020
S75	[104]	Deep Learning Framework for Face Mask Detection	Face detection model for face mask detection in video streams	ICOEI	2021
S76	[105]	Deep Learning Implementation of Facemask Detection	Real-time mask detection method	CONFCDS	2021
S77	[106]	Deployment of a smart handwashing station in a school setting during the COVID-19 pandemic: Field study	Smart handwashing station	JMIR	2020
S78	[107]	Design and Evaluation of a System for Decentralized Management of Solidarity Actions during the COVID-19 Crisis	Web system for Decentralized Management of Solidarity Actions during the COVID-19 Crisis	Appl. Sci.	2020
S79	[108]	Design and implementation of a social distancing and contact tracing wearable	Implementation of a social distancing and contact tracing wearable	CiSt	2020
S80	[109]	Design of hexacopter UAV system for disinfectant spraying	UAV system as a disinfectant sprayer with drone adoption	EIC	2020
S81	[110]	Design of Infectious Disease Prevention and Control Platform Based on Big Data Analysis of Location Information	Big data analysis platform for individual positioning information	EITCE	2020
S82	[111]	Design of Smart-Gate Based on Artificial Intelligence Possibly for COVID-19 Early Prevention at Public Area	Artificial intelligence-based smart gate and website integration	TSSA	2020
S83	[112]	Designing a Multi-Agent Occupant Simulation System to Support Facility Planning and Analysis for COVID-19	Multi-agent occupancy simulation system that helps people to understand and evaluate risks associated with virus transmission	DIS	2021
S84	[113]	Designing for pandemics—A design concept based on technology mediated nudging for health behavior change	Application that promotes health behavior change based on Bluetooth proximity estimation and nudging theory	HICSS	2021
S85	[114]	Detecting Spatial Clusters of Disease Infection Risk Using Sparsely Sampled Social Media Mobility Patterns	New spatial scan methods to search for spatial clusters of increased infection risk	SIGSPATIAL	2019
S86	[115]	Detection and monitoring of the asymptotic COVID-19 patients using IoT devices and sensors	Detect and monitor the asymptotic patients using IoT-based sensors	IJPCC	2020
S87	[116]	Detection and Tracking Contagion using IoT-Edge Technologies: Confronting COVID-19 Pandemic	Smart edge surveillance system to help to detect and track the contagious person	ICECCE	2020
S88	[117]	Detection of COVID-19 in Chest X-ray Image using CLAHE and Convolutional Neural Network	COVID-19 detection in Chest X-ray Images	ICORIS	2020
S89	[118]	Detection of SARS-CoV-2 in the air of two hospitals in Hermosillo, Sonora, México, utilizing a low-cost environmental monitoring system	Filtration system with vacuum pump for detecting the COVID-19 virus	IJID	2021
S90	[119]	Developing Smart COVID-19 Social Distancing Surveillance Drone using YOLO Implemented in Robot Operating System simulation environment	COVID-19 Social Distancing Surveillance system	R10-HTC	2020
S91	[120]	Development and application of agent-based disease spread simulation model: the case of suwon, Korea	Agent-based disease diffusion model that reflects the nature of the disease and the structural and statistical characteristics of population	WSC	2017
S92	[121]	Development of The Personnel Monitoring System Using Mobile Application and Real-Time Database During the COVID19 Pandemic	Real-time monitoring and command system using mobile applications and cloud computing technology	ISRITI	2020
S93	[122]	Digital health technologies respond to the COVID-19 pandemic in a tertiary hospital in China: Development and usability study	Web-based COVID-19 service platform	JMIR	2020
S94	[123]	Digital proximity tracing on empirical contact networks for pandemic control	Framework informed by empirical high-resolution contact data to analyze the impact of digital contact tracing in the COVID-19 pandemic	Nat. Commun.	2021
S95	[124]	Disinfection of Klebsiella pneumoniae using ultrasonic systems	Disinfection of Klebsiella pneumoniae using ultrasonic systems	J. Environ. Biol.	2016
S96	[125]	Dynamic multiplex social network models on multiple time scales for simulating contact formation and patterns in epidemic spread	Model for dynamic networks of physical contacts and their application for reproducing complex patterns in epidemic spread	WSC	2017
S97	[126]	Dynamical SEIR Model with Information Entropy Using COVID-19 as a Case Study	SEIR model that incorporates social media into disease spread dynamics, and allow epidemic spread simulation to facilitate prediction	IEEE Trans. Comput. Soc. Syst.	2021
S98	[127]	Economic Evaluation of Quarantine: A Case Study of COVID-19 Pandemic in Belgium	SEIR-based simulation model to predict the disease transmission	ICIIBMS	2020
S99	[128]	Effective real-time allocation of pandemic interventions	Agent based model for simulating the diffusion of pandemic influenza via carefully calibrated inter-city airline travel	WSC	2010
S100	[129]	E-health: agent-based models to simulate behavior of individuals during an epidemic outbreak	Agent based model of behavior and activities of individuals	DG.O	2018
S101	[130]	Epidemic Propagation with Positive and Negative Preventive Information in Multiplex Networks	Epidemic model to explore the characteristics of epidemic propagation under the impact of positive and negative prevention information	IEEE Trans Cybern	2021
S102	[131]	EpiSimdemics: an efficient algorithm for simulating the spread of infectious disease over large realistic social networks	Scalable parallel algorithm to simulate the spread of contagion in large, realistic social contact networks	SC	2008
S103	[132]	Exploiting Social Media to enhance Clinical Decision Support	Clinical Decision Support System to assist diagnosing or treating patients	WI Companion	2019
S104	[133]	Exploring Automatic Diagnosis of COVID-19 from Crowdsourced Respiratory Sound Data	Crowdsource respiratory sounds and study how such data may aid COVID-19 diagnosis	KDD	2020
S105	[134]	Extraction of disease events for a real-time monitoring system	Semantic rules and machine learning adoption to extract infectious disease events	SoICT	2014
S106	[135]	Face Mask Detection using Convolutional Neural Network (CNN) to reduce the spread of COVID-19	Deep learning methods adoption for face mask detection	ICOEI	2021
S107	[136]	Face Mask Detection Using Deep Learning	Deep learning methods adoption for face mask detection	ICCCI	2021
S108	[137]	Face mask detection using YOLOv3 and faster R-CNN models: COVID-19 environment	Face mask detection using YOLOv3 and faster R-CNN models	Multimed Tools Appl	2021
S109	[138]	FaceLock Homes: A Contactless Smart Home Security System to Prevent COVID Transmission	IoT adoption to unauthorized access restriction system in home security, and human screening check for COVID transmission prevention	WiSPNET	2021
S110	[139]	Facing COVID-19 pandemic: Development of custom-made face mask with rapid prototyping system	3D printing technology adoption for personal protective equipment construction for healthcare professionals	JIDC	2021
S111	[140]	Fast Containment of Infectious Diseases with E-healthcare Mobile Social Internet of Things	Healthcare mobile social internet of things based targeted vaccination scheme to fast contain the spread of the infectious disease	IEEE Internet Things J	2021
S112	[141]	Fine-Tuning A Lightweight Convolutional Neural Networks for COVID-19 Diagnosis	Deep learning adoption s for automatic COVID-19 diagnosis in chest x-ray image datasets	CSBio	2020
S113	[142]	First prototype of the infectious diseases seeker (IDS) software for prompt identification of infectious diseases	Infectious disease regressive prediction tool	JEGH	2020
S114	[143]	FluSense: A Contactless Syndromic Surveillance Platform for Influenza-Like Illness in Hospital Waiting Areas	Contactless syndromic surveillance platform FluSens	IMWUT	2020
S115	[144]	Fluspot: Seasonal flu tracking app exploiting wearable IoT device for symptoms monitoring	Application development and IoT adoption to contribute to virus prevention	SEEDA-CECNSM	2020
S116	[145]	Forecasting Seasonal Influenza Fusing Digital Indicators and a Mechanistic Disease Model	Seasonal influenza forecast framework based on a stochastic, spatially structured mechanistic model	WWW Companion	2017
S117	[146]	GeoAI-based Epidemic Control with Geo-Social Data Sharing on Blockchain	Infectious disease information sharing platform by combining the Blockchain, social apps, and Geospatial Artificial Intelligence	HEALTHCOM	2021
S118	[147]	Handheld plasmonic biosensor for virus detection in field-settings	Biosensor that employs a plasmonic chip based on nanohole arrays integrated to a lensfree-imaging framework for label-free detection of viruses	Sens. Actuators	2021
S119	[148]	Heuristic Evaluation of an African-centric Mobile Persuasive Game for Promoting Safety Measures Against COVID-19	Mobile game aimed at raising awareness on the importance of social distancing and other precautionary measures	AfriCHI	2021
S120	[149]	Hi-COVIDNet: Deep Learning Approach to Predict Inbound COVID-19 Patients and Case Study in South Korea	Novel approach to address the problem of predicting imported COVID-19 cases	KDD	2020
S121	[150]	Human Face Recognition and Temperature Measurement Based on Deep Learning for COVID-19 Quarantine Checkpoint	Method that combines body temperature measurement, facial recognition, and masking based on deep learning	ICFNDS	2020
S122	[151]	Human Temperature Scanning from a Distance	Solution of measuring human temperature from a distance	CSCI	2020
S123	[152]	IDispenser-big data enabled intelligent dispenser	Approach to prevent spread of airborne diseases through the application of Big Data Technologies and IoT Sensing	BigDataService	2017
S124	[153]	Improving infectious diseases prevention system: The case study of Departement of Health Sragen	Early warning system for infectious diseases prevention	ICITSI	2015
S125	[154]	Individual Decision Making Can Drive Epidemics: A Fuzzy Cognitive Map Study	Fuzzy cognitive map (FCM) denotative model to consider individual behavior and its influence on individual decision making to prevent infections	IEEE Trans Fuzzy Syst	2013
S126	[155]	Indoor air quality monitoring system for proactive control of respiratory infectious diseases: poster abstract	Low-cost indoor air quality monitoring devices and systems to tackle the disease surveillance problem	SenSys	2020
S127	[156]	Infection Prevention and Control using UV-Disinfectant Bot for COVID	Infection prevention method and Ultraviolet rays disinfection robots adoption for hospital sterilization	INCET	2021
S128	[157]	Infection Screening System Using Thermography and CCD Camera with Good Stability and Swiftness for Non-contact Vital-Signs Measurement by Feature Matching and MUSIC Algorithm	Infectious diseases screening system using feature matching and MUSIC algorithm	EMBC	2019
S129	[158]	Infectious Diseases Spreading on an Adaptive Metapopulation Network	Adaptive metapopulation network and risk indicator according to the relative infection density definition	IEEE Access	2020
S130	[159]	Inferring Metapopulation Propagation Network for Intra-city Epidemic Control and Prevention	Two-step method for intra-city epidemic propagation modeling on a metapopulation base	KDD	2018
S131	[160]	Initial development of “Infection defender”: A children’s educational game for pandemic prevention measurements	Game to promote children’s awareness in spread of infectious diseases prevention measurements	VISIGRAPP	2021
S132	[161]	Integrated agent-oriented modeling and simulation of population and healthcare delivery network: application to COPD chronic disease in a Canadian region	Framework for integrated agent-oriented modeling and simulation of the population with a specific chronic disease	WSC	2010
S133	[162]	Intelligent Lighting System Design Based on UV LED Technology with the Functions of Air Sterilization, Disinfection, Purification and Mosquito Control	Integration of air elimination, purification, and mosquito control into commonly used lighting fixtures	SSLChina: IFWS	2020
S134	[163]	IoT based COVID De-Escalation System using Bluetooth Low Level Energy	Monitor real time cases and prevent the contagious spread of COVID	ICICT	2021
S135	[164]	IoT Telemonitoring System for COVID-19 Quarantine	IoT telemonitoring system for quarantine	TEM	2021
S136	[165]	IoT-based GPS assisted surveillance system with inter-WBAN geographic routing for pandemic situations	Surveillance system with IoT applications and an inter-WBAN geographic routing algorithm	JBI	2021
S137	[166]	IVACS: Intelligent Voice Assistant for Coronavirus Disease (COVID-19) Self-Assessment	Intelligent voice-based assistant for COVID-19 self-assessment	ICAIMAT	2020
S138	[167]	Link Analysis to Discover Insights from Structured and Unstructured Data on COVID-19	Mining method to answer the call to action and help the science community answer high-priority scientific questions related to SARS-CoV-2	BCB	2020
S139	[168]	Longitudinal remotely mentored self-performed lung ultrasound surveillance of paucisymptomatic COVID-19 patients at risk of disease progression	Remotely tele mentored self-performed ultrasound to monitor people with COVID-19 risk progression	J. Ultrasound	2021
S140	[169]	Low-cost Robot Assistance Design for Health Area to Help Prevent COVID-19 in Honduras	Robots design to perform simple tasks in hospitals	ICRAI	2020
S141	[170]	Machine learning framework for COVID-19 diagnosis	Machine learning application for early diagnosis of disease and continuous monitoring of infected patients	DATA	2021
S142	[171]	Mask Detection and Epidemic Prevention System	Face mask recognition and epidemic prevention system	ICMSP	2020
S143	[172]	Mask Wearing Classification using CNN	Deep learning based image classification model to detect whether a person is wearing a mask or not	ICAICTA	2020
S144	[173]	Mask wearing detection method based on SSD-Mask algorithm	Method of face mask wearing detection in natural scenes	ICCSMT	2020
S145	[174]	MAS-SEIR-II Simulation on COVID-19 in China	Model based on regional geographic information to hypothetically simulate different epidemic prevention and control scenarios	ICBDM	2020
S146	[175]	Mathematical analysis of an SIR network model with imperfect vaccination and varying size of population	Modified susceptible-infected-recovered model on homogeneous networks regarding epidemic spreading	ICCMS	2017
S147	[176]	Measles Metapopulation Modeling using Ideal Flow of Transportation Networks	Big data adoption in the monitoring of measles propagation	ICSIM	2019
S148	[177]	Medical imaging engineering and technology branch of the chinese society of biomedical engineering expert consensus on the application of emergency mobile cabin CT	Emergency mobile cabin CT for scan and diagnostic	Quant Imaging Med Surg	2020
S149	[178]	Mining Spatiotemporal Diffusion Network: A New Framework of Active Surveillance Planning	Framework of active surveillance planning of infectious disease spread	IEEE Access	2019
S150	[179]	Mobile support for diagnosis of communicable diseases in remote locations	Mobile system adoption to assist health professionals with diagnosis of emerging and neglected diseases	CHINZ	2012
S151	[180]	Mobile tools for point-of-care diagnostics in the developing world	Smartphone application for rapid diagnostic tests	ACM DEV	2013
S152	[181]	Mobilenet mask: A multi-phase face mask detection model to prevent person-to-person transmission of SARS-CoV-2	Deep learning-based multi-phase face mask detection model	TCCE	2020
S153	[182]	Mobility-guided modeling of the COVID-19 pandemic in Metro Manila	Model to simulate the pandemic	PJS	2020
S154	[183]	Modeling and mining spatiotemporal patterns of infection risk from heterogeneous data for active surveillance planning	Method to active surveillance planning via modeling and mining spatiotemporal patterns of infection risks from heterogeneous data sources	AAAI	2014
S155	[184]	Modeling Individual-Level Infection Dynamics Using Social Network Information	Non-invasive disease monitoring system that accurately identifies the specific people who are sick, without explicitly diagnosing them	CIKM	2015
S156	[185]	Modelling the response of a public health department to infectious disease	Discrete-event simulation model of the response of a local public health department to pertussis cases	WSC	2010
S157	[186]	Monitoring health care workers at risk for COVID-19 using wearable sensors and smartphone technology: Protocol for an observational mHealth study	Physiological data monitoring of healthcare workers	JMIR	2021
S158	[187]	Multilingual COVID-QA: Learning towards Global Information Sharing via Web Question Answering in Multiple Languages	Multilingual COVID-QA model to answer people’s questions in their own languages	WWW Companion	2021
S159	[188]	NanoSPC: A scalable, portable, cloud compatible viral nanopore metagenomic data processing pipeline	Pipeline for analyzing Nanopore sequencing data to identify potentially pathogenic viruses and bacteria	NAR	2020
S160	[189]	On calibrating a microsimulation of patient movement through a healthcare network	Microsimulation to simulate pathogen transmission among individuals	WSC	2019
S161	[190]	Optimal control strategy for a multi-regional epidemic model	Multi-regional model for the global spread of an emerging and re-emerging infectious disease	WCICA	2012
S162	[191]	P2B-Trace: Privacy-Preserving Blockchain-based Contact Tracing to Combat Pandemics	P2B-Trace, a privacy-preserving contact tracing initiative based on blockchain	SIGMOD	2021
S163	[192]	Pandemic Stabilizer using Smartwatch	Smartwatch adoption to screen individuals for body temperature, heart rate and blood pressure	DASA	2020
S164	[193]	Parallel low discrepancy parameter sweep for public health policy	Approach that pre-computes simulations of passenger movement, performing only the disease-specific analysis in real time	CCGrid	2018
S165	[194]	Participatory disease detection through digital volunteerism: how the doctorme application aims to capture data for faster disease detection in thailand	Disease Detection through Digital Volunteerism and Mobile application adoption	WWW Companion	2014
S166	[195]	People Under Surveillance Tracker Prototype Development with Bluetooth Low Energy to Suppress the COVID-19 Spread	Mobile-based tracking application	ICORIS	2020
S167	[196]	Physical distancing and risk of COVID-19 in small-scale fisheries: a remote sensing assessment in coastal Ghana	Unmanned Aerial Vehicle technology adoption for policy direction and intervention in pandemic	Sci Rep	2020
S168	[197]	Potential application of novel technology developed for instant decontamination of personal protective equipment before the doffing step	Spray disinfection technology (chamber)	PLoS ONE	2021
S169	[198]	Predicting COVID-19 infections and deaths in Bangladesh using Machine Learning Algorithms	Prediction of COVID-19 pandemic future cases by the adoption and exploration of machine learning algorithms	ICICT4SD	2021
S170	[199]	Prediction on COVID-19 via Logit Model for the Five Worst-Affected Countries in Global	Prediction on COVID-19 spread via Logit Model	CONFCDS	2021
S171	[200]	Prevention from COVID-19 in India: Fuzzy Logic Approach	Fuzzy Logic approach application to identify possible infections by symptom analysis	ICACITE	2021
S172	[201]	Privacy-preserving People Counting with Channel State Information	Method of counting the number of people in the space	ICTC	2020
S173	[202]	Private blockchain-envisioned security framework for AI-enabled IoT-based drone-aided healthcare services	Private-blockchain based framework for secure communication in an IoT-enabled drone-aided healthcare environment	DroneCom	2020
S174	[203]	Protecting healthcare workers during COVID-19 pandemic with nanotechnology: A protocol for a new device from Egypt	Design for antimicrobial and antiviral respirator facial mask with nanotechnology	J. Infect. Public Health	2020
S175	[204]	Proximity tracing method to reduce community spread of COVID 19	Mobile app wherein a user who is infected or detected with corona positive can be backward traced	JET	2020
S176	[205]	Rapid deployment of a free, privacy-assured COVID-19 symptom tracker for public safety during reopening: System development and feasibility study	Develop a monitoring and reporting system for COVID-19 to support institutions conducting monitoring activities	JMIR	2020
S177	[206]	Realization of Temperature Measurement by Passive Terahertz Imaging	Temperature Measurement by Passive Terahertz Imaging	UCMMT	2020
S178	[207]	Real-time Mask Identification for COVID-19: An Edge Computing-based Deep Learning Framework	ECMask—Edge computing-based mask identification framework	IEEE Internet Things J	2021
S179	[208]	Rebirth-20—Relive After the COVID-19 and Keep Preventing Against it	Software for teaching gestures and actions that contribute to the prevention of COVID and also provide exercises for recovery after contracting COVID	VRW	2021
S180	[209]	Recommendation rules mining for reducing the spread of COVID-19 cases	Recommendation rules building for appropriated state policy for reducing the spread of new COVID-19 cases	IDDM	2020
S181	[210]	Remote sensing of vital signs: A Wearable, Wireless “band-Aid” sensor with personalized analytics for improved ebola patient care and worker safety	Wearable, Wireless “band-Aid” sensor with personalized analytics for improved Ebola patient care and worker safety	GHSP	2015
S182	[211]	RFWash: a weakly supervised tracking of hand hygiene technique	Radio-based device-free system for monitoring Hand Hygiene technique	SenSys	2020
S183	[212]	Role of 5G-powered remote robotic ultrasound during the COVID-19 outbreak: Insights from two cases	Robotic ultrasound based on 5G-powered technology adoption	Eur Rev Med Pharmacol Sci	2020
S184	[213]	SD-Measure: A Social Distancing Detector	Novel framework for detecting social distancing from video footages	CICN	2020
S185	[214]	Secured College Bus Management System using IoT for COVID-19 Pandemic Situation	Secured College Bus Management System (SCBMS) for student screening and mask detection	ICICV	2021
S186	[215]	Security Control Components for Epidemic Prevention Donation Management Blockchain	Blockchain adoption in order to improve security in information control of donations management in pandemic scenarios	BSCI	2020
S187	[216]	Seeing is Comforting: Effects of Teleoperator Visibility in Robot-Mediated Health Care	Robot teleoperator adoption in patients’ medical care	HRI	2016
S188	[217]	Simulating the Evolution of Infectious Agents Through Human Interaction	Software simulation method for spread prevention	SIITME	2020
S189	[218]	Simulation Analysis of Epidemic Trend for COVID-19 Based on SEIRS Model	Model to simulate and forecast the trend of COVID-19 epidemic	ICCI*CC	2020
S190	[219]	Simulation of strategies for containing pandemic influenza	Stochastic simulation model of pandemic influenza to investigate realistic intervention strategies in reaction to developing outbreaks	WSC	2010
S191	[220]	Smart epidemic tunnel: IoT-based sensor-fusion assistive technology for COVID-19 disinfection	IoT-based sensor-fusion assistive technology for COVID-19 disinfection	IJPCC	2020
S192	[221]	Smart homes that detect sneeze, cough, and face touching	Smart home monitoring system to detect coughing, sneezing, face touching, and entering/leaving a room	Smart Health	2021
S193	[222]	SmartGate system: Automating the detection and control of COVID-19	SmartGate to detect the potential of the virus in patients without human intervention	ICFNDS	2020
S194	[223]	Social Distancing using Bluetooth Low Energy to Prevent the Spread of COVID-19	Development of an Android Application for Social Distancing Alert System	Confluence	2021
S195	[224]	Social distancing warning system at public transportation by analyzing wi-fi signal from mobile devices	Social distancing warning system	ISWC	2020
S196	[225]	SociTrack: infrastructure-free interaction tracking through mobile sensor networks	Platform for autonomous social interaction tracking via wireless distance measurements	MobiCom	2020
S197	[226]	Solar Power based Intelligent System for Hand wash cum Dryer to Conflict the Outbreak of COVID-19	Solar Powered automatic hand dryer system	ICISS	2020
S198	[227]	Sonography of the Lungs: Diagnosis and Surveillance of Patients With COVID-19	Use of sonography to evaluate the lungs for COVID-19 infection identification	JDMS	2020
S199	[228]	System design of safety road network in urban morphology prevention during COVID-19 based on digital simulation technology	System design of public transportation, prevention, and control of urban pandemics	BDE	2020
S200	[229]	Targeted Vaccination for COVID-19 Using Mobile Communication Networks	Targeted vaccination mathematical method to allocate a limited number of vaccines	IKT	2020
S201	[230]	TDEFSI: Theory-guided Deep Learning-based Epidemic Forecasting with Synthetic Information	Theory-guided Deep Learning-based Epidemic Forecasting	TSAS	2020
S202	[231]	Telehealth mask wearing training for children with autism during the COVID-19 pandemic	Telehealth mask wearing training for children with autism	J. Appl. Behav. Anal.	2021
S203	[232]	The Application of Mobile Telehealth System to Facilitate Patient Information Presentation and Case Discussion	Mobile Telehealth System to Facilitate Patient Information Presentation and Case Discussion	Telemed E-Health	2020
S204	[233]	The development of the geographical information system (GIS)-based mapping of infectious diseases using spatial data analysis	Web-based Decision Support System with Android Mobile Support, Mapping and Short Message Service development and adoption	IJATCSE	2019
S205	[234]	The effectiveness of disinfectant spraying based on drone technology	Disinfectant spraying application and testing based on drone technology	EIC	2020
S206	[235]	The Impact of Histogram Equalization and Color Mapping on ResNet-34’s Overall Performance for COVID-19 Detection	Methodology to assess the overall performance of a deep CNN architecture for COVID-19 detection	DSDE	2021
S207	[236]	The Implementation of an Emergency Medicine Telehealth System During a Pandemic	Emergency medicine telehealth system implementation	JEM	2021
S208	[237]	The picture of health: map-based, collaborative spatio-temporal disease tracking	System adoption for disease detection and tracking by geotagging ProMED-mail postings	HealthGIS	2012
S209	[238]	The study of colleges students returning to campus under the epidemic situation based on GIS	Formulate a strategy for college students returning to campus in batches after the epidemic	EMGIS	2020
S210	[239]	To quarantine, or not to quarantine: A theoretical framework for disease control via contact tracing	Compartmental model for COVID19 disease progression, in a modeling framework that captures testing and digital contact tracing	Epidemics	2021
S211	[240]	Toward optimal resource-allocation for control of epidemics: an agent-based-simulation approach	Agent-based simulation model of epidemics	WSC	2010
S212	[241]	Tracking and visualization of space-time activities for a micro-scale flu transmission study	Use of tracking devices to collect data of space-time trajectories and the spatiotemporal processing of such data to facilitate flu transmission study	IJHG	2013
S213	[242]	Tracking Urban Mobility and Occupancy under Social Distancing Policy	Multi-scale map of urban mobility and space occupancy under social distancing policy	DGOV	2020
S214	[243]	Tracy: Smartphone-based Contact Tracing Solution that Supports Self-investigation to Limit the Spread of COVID-19	innovative privacy preserving smartphone-based contact tracing solution	NILES	2020
S215	[244]	Two Approximate Dynamic Programming Algorithms for Managing Complete SIS Networks	Dynamic Programming Algorithms for Managing Complete SIS Networks inspired by the case study of mosquito Aedes albopictus	COMPASS	2018
S216	[245]	Understanding the Urban Pandemic Spreading of COVID-19 with Real World Mobility Data	Data-driven epidemic simulator with COVID-19 specific features	KDD	2020
S217	[246]	Using a sharing-platform to prevent a new outbreak of COVID-19 pandemic in rural areas	Develop an exchange platform to track the spread of COVID-19 in rural areas	GJESM	2021
S218	[247]	Using machine vision for functionality expansion of mini robots decontaminating medical personnel premises in conditions of COVID-19 epidemic	Mobile autonomous robots’ adoption to reduce risks of infections of medical personnel	ISPRS Archives	2021
S219	[248]	Wireless applications for hospital epidemiology	Low-cost wireless system to instrument hand-hygiene events	WiMD	2019

**Table A2:** Mapping of studies regarding digital libraries.

ID	ACM	IEEE	Scopus	Compendex	ID	ACM	IEEE	Scopus	Compendex	ID	ACM	IEEE	Scopus	Compendex	ID	ACM	IEEE	Scopus	Compendex
S1	X				S56			X	X	S111		X			S166		X		
S2	X				S57		X			S112	X				S167			X	
S3		X	X	X	S58		X			S113			X		S168			X	
S4	X				S59	X				S114	X				S169		X		
S5	X	X			S60	X				S115		X	X	X	S170	X			
S6		X			S61		X			S116	X				S171		X		
S7		X			S62	X				S117		X			S172		X		
S8	X				S63		X			S118			X	X	S173	X			
S9	X				S64	X				S119	X				S174			X	
S10		X			S65		X	X	X	S120	X				S175			X	
S11			X		S66	X				S121	X				S176			X	
S12		X	X	X	S67		X	X	X	S122		X			S177		X	X	X
S13			X		S68		X	X	X	S123		X	X		S178		X		
S14	X				S69			X	X	S124		X	X	X	S179		X		
S15		X			S70	X				S125		X			S180			X	
S16		X	X		S71		X	X	X	S126	X				S181			X	
S17	X	X			S72			X	X	S127		X			S182	X			
S18	X				S73		X			S128		X			S183			X	
S19	X				S74			X	X	S129		X			S184		X		
S20		X			S75		X			S130	X				S185		X		
S21		X			S76	X				S131				X	S186	X			
S22		X			S77			X		S132	X				S187	X	X		
S23	X				S78			X		S133		X	X	X	S188		X		
S24			X		S79		X	X		S134		X	X	X	S189		X		
S25			X		S80			X	X	S135			X		S190	X			
S26			X	X	S81	X				S136			X	X	S191			X	
S27			X		S82		X			S137		X			S192			X	
S28	X				S83	X				S138	X				S193	X		X	
S29	X				S84			X	X	S139			X		S194		X	X	X
S30		X			S85	X				S140	X				S195	X			
S31		X			S86			X		S141	X		X		S196	X			
S32		X			S87		X	X		S142			X	X	S197		X	X	
S33	X				S88		X	X	X	S143		X			S198			X	
S34			X	X	S89			X		S144		X			S199	X		X	X
S35				X	S90	X				S145	X				S200		X		
S36	X				S91	X	X			S146	X				S201	X			
S37	X				S92		X	X	X	S147	X				S202			X	
S38		X			S93			X		S148			X		S203			X	X
S39			X		S94			X		S149		X			S204			X	
S40	X				S95			X		S150	X				S205			X	X
S41	X				S96	X	X			S151	X				S206	X			
S42			X		S97		X			S152			X	X	S207			X	
S43	X				S98		X			S153			X		S208	X			
S44	X				S99	X				S154	X			X	S209	X			
S45			X		S100	X				S155	X				S210			X	
S46	X				S101		X			S156	X				S211	X			
S47		X	X	X	S102	X	X			S157			X		S212			X	
S48	X				S103	X	X			S158	X				S213	X			
S49		X	X	X	S104	X				S159			X		S214		X	X	
S50			X	X	S105	X				S160	X	X			S215	X			
S51			X		S106		X			S161		X			S216	X			
S52		X			S107		X			S162	X				S217			X	
S53	X				S108			X	X	S163		X	X		S218			X	
S54			X		S109		X			S164	X	X			S219	X			
S55		X			S110			X		S165	X								

**Table A3:** Distribution of selected papers on publication venues.

Publication Venue	#	%	Publication Venue	#	%	Publication Venue	#	%	Publication Venue	#	%
WSC	10	4.57	CICN	1	0.46	ICCTIDE	1	0.46	JEM	1	0.46
JMIR	4	1.82	CICT	1	0.46	ICECCE	1	0.46	JET	1	0.46
KDD	4	1.82	CiSt	1	0.46	ICICT	1	0.46	JGE	1	0.46
WWW Companion	4	1.82	CoCoNet	1	0.46	ICICV	1	0.46	JIDC	1	0.46
CONFCDS	3	1.37	COMPASS	1	0.46	ICIIBMS	1	0.46	Methods Inf. Med.	1	0.46
IEEE Access	3	1.37	Confluence	1	0.46	ICISA	1	0.46	Mob. Netw. Appl.	1	0.46
IEEE Internet Things J.	3	1.37	CSBio	1	0.46	ICISS	1	0.46	Mobidata	1	0.46
SIGSPATIAL	3	1.37	CSCI	1	0.46	ICITSI	1	0.46	MobiWac	1	0.46
ISWC	2	0.91	CVIDL	1	0.46	ICMSP	1	0.46	Multimed Tools Appl	1	0.46
ICMHI	2	0.91	DASA	1	0.46	ICPECTS	1	0.46	NAR	1	0.46
CIKM	2	0.91	DATA	1	0.46	ICPS	1	0.46	Nat. Commun.	1	0.46
DG.O	2	0.91	DEST	1	0.46	ICRAI	1	0.46	NILES	1	0.46
DGOV	2	0.91	DH	1	0.46	ICSIM	1	0.46	Pers. Ubiquitous Comput.	1	0.46
EIC	2	0.91	DIS	1	0.46	ICTC	1	0.46	PJS	1	0.46
Healthcom	2	0.91	DroneCom	1	0.46	ICUIMC	1	0.46	PLoS ONE	1	0.46
ICAIS	2	0.91	DSAI	1	0.46	IDDM	1	0.46	Quant Imaging Med Surg	1	0.46
ICCCS	2	0.91	DSDE	1	0.46	IEEE J Biomed Health Inform	1	0.46	R10-HTC	1	0.46
ICFNDS	2	0.91	DSIT	1	0.46	IEEE Trans Cybern	1	0.46	SC	1	0.46
ICICT4SD	2	0.91	EATIS	1	0.46	IEEE Trans Fuzzy Syst	1	0.46	SCA	1	0.46
ICOEI	2	0.91	EITCE	1	0.46	IEEE Trans. Big Data	1	0.46	Sci Rep	1	0.46
ICORIS	2	0.91	EMGIS	1	0.46	IEEE Trans. Comput. Soc. Syst.	1	0.46	SEEDA-CECNSM	1	0.46
IJPCC	2	0.91	EMBS	1	0.46	IJACSA	1	0.46	Sens. Actuators	1	0.46
MobiCom	2	0.91	Epidemics	1	0.46	IJATCSE	1	0.46	SIGIR	1	0.46
SenSys	2	0.91	Eur Rev Med Pharmacol Sci	1	0.46	IJCRR	1	0.46	SIGMOD	1	0.46
SoICT	2	0.91	Front. Robot. AI	1	0.46	IJDSN	1	0.46	SIITME	1	0.46
WebSci	1	0.46	FRUCT	1	0.46	IJERPH	1	0.46	Smart Health	1	0.46
AAAI	1	0.46	GCAIoT	1	0.46	IJHG	1	0.46	Socio-Econ. Plan. Sci.	1	0.46
Appl. Sci.	1	0.46	GHSP	1	0.46	IJID	1	0.46	SSLChina: IFWS	1	0.46
ACM DEV	1	0.46	GJESM	1	0.46	IKT	1	0.46	TCBB	1	0.46
AfriCHI	1	0.46	HICSS	1	0.46	IMWUT	1	0.46	TCCE	1	0.46
EMBC	1	0.46	HRI	1	0.46	INCET	1	0.46	TCSS	1	0.46
AIID	1	0.46	ICACCS	1	0.46	IoT Companion	1	0.46	Telemed E-Health	1	0.46
Arab J Sci Eng.	1	0.46	ICACIE	1	0.46	ISCTT	1	0.46	TEM	1	0.46
ARES	1	0.46	ICACITE	1	0.46	ISPRS Archives	1	0.46	TMIS	1	0.46
HealthGIS	1	0.46	ICAICTA	1	0.46	ISRITI	1	0.46	TSAS	1	0.46
BCB	1	0.46	ICAIMAT	1	0.46	J. Appl. Behav. Anal.	1	0.46	TSSA	1	0.46
BDE	1	0.46	ICASIT	1	0.46	J. Environ. Biol.	1	0.46	UCMMT	1	0.46
BigDataService	1	0.46	ICBDM	1	0.46	J. Infect. Public Health	1	0.46	UEMCON	1	0.46
BSCI	1	0.46	ICCCI	1	0.46	J. Intell. Fuzzy Syst.	1	0.46	VISIGRAPP	1	0.46
CAADRIA	1	0.46	ICCE-Taiwan	1	0.46	J. Ultrasound	1	0.46	VRW	1	0.46
CCEAI	1	0.46	ICCI	1	0.46	JBI	1	0.46	WCICA	1	0.46
CCGrid	1	0.46	ICCI*CC	1	0.46	JBSBE	1	0.46	WI Companion	1	0.46
CCWC	1	0.46	ICCMS	1	0.46	JDMS	1	0.46	WiMD	1	0.46
CHINZ	1	0.46	ICCSMT	1	0.46	JEGH	1	0.46	WiSPNET	1	0.46

**Table A4:** Citation counts overview.

ID	Citation Count	ID	Citation Count	ID	Citation Count	ID	Citation Count	ID	Citation Count	ID	Citation Count	ID	Citation Count	ID	Citation Count	ID	Citation Count
S102	338	S12	13	S210	7	S152	3	S21	1	S215		S60	0	S115	0	S170	0
S5	138	S29	13	S17	6	S153	3	S22	1	S1	0	S63	0	S117	0	S171	0
S104	82	S123	13	S36	6	S160	3	S25	1	S2	0	S65	0	S118	0	S177	0
S116	64	S150	13	S48	6	S182	3	S30	1	S8	0	S66	0	S119	0	S179	0
S101	45	S155	13	S184	6	S183	3	S61	1	S10	0	S69	0	S121	0	S186	0
S212	38	S165	13	S198	6	S196	3	S68	1	S11	0	S70	0	S122	0	S188	0
S114	37	S49	12	S28	5	S7	2	S71	1	S13	0	S72	0	S124	0	S189	0
S125	37	S154	12	S46	5	S34	2	S73	1	S14	0	S74	0	S126	0	S193	0
S208	28	S201	12	S96	5	S57	2	S82	1	S16	0	S75	0	S127	0	S194	0
S132	27	S9	11	S96	5	S59	2	S95	1	S24	0	S76	0	S131	0	S195	0
S211	22	S202	11	S134	5	S64	2	S100	1	S27	0	S78	0	S133	0	S197	0
S174	20	S19	10	S159	5	S67	2	S110	1	S31	0	S79	0	S135	0	S199	0
S187	20	S94	10	S41	4	S77	2	S111	1	S32	0	S80	0	S138	0	S200	0
S4	19	S167	10	S43	4	S91	2	S129	1	S35	0	S81	0	S140	0	S204	0
S151	18	S52	9	S105	4	S103	2	S137	1	S37	0	S83	0	S141	0	S205	0
S191	18	S120	8	S149	4	S113	2	S139	1	S38	0	S84	0	S142	0	S206	0
S26	17	S156	8	S176	4	S136	2	S146	1	S39	0	S88	0	S143	0	S209	0
S181	17	S164	8	S190	4	S147	2	S163	1	S42	0	S90	0	S144	0	S217	0
S130	16	S178	8	S216	4	S162	2	S168	1	S45	0	S92	0	S145	0	S218	0
S23	15	S51	7	S20	3	S180	2	S172	1	S47	0	S97	0	S148	0		
S40	15	S85	7	S44	3	S185	2	S175	1	S50	0	S98	0	S157	0		
S86	15	S87	7	S58	3	S3	1	S192	1	S53	0	S106	0	S158	0		
S219	15	S99	7	S62	3	S6	1	S207	1	S54	0	S107	0	S161	0		
S33	14	S128	7	S89	3	S15	1	S213	1	S55	0	S109	0	S166	0		
S203	14	S173	7	S108	3	S18	1	S214	1	S56	0	S112	0	S169	0		

**Table A5:** Publications distributed by countries and application domain category.

Countries/ Categories	CD1	CD2	CD3	CD4	CD5	CD6
China (48)	S58, S60, S61, S133, S158, S183, S203	S26, S148, S177, S198	S6, S8, S76, S142, S144, S178	S15, S17, S32, S41, S45, S55, S66, S81, S97, S98, S101, S111, S117, S125, S129, S130, S145, S146, S161, S170, S186, S189, S199, S209, S216	S50	S34, S49, S149, S154, S162
USA (38)	S4, S93, S123, S137, S151, S157, S160, S181, S187, S192, S207, S219	S122		S5, S28, S29, S46, S48, S53, S62, S71, S99, S102, S116, S138, S155, S156, S164, S201, S211	S16, S69, S213	S114, S176, S196, S208, S212
India (31)	S11, S27, S31, S37, S38, S57, S127, S141, S171, S191	S163	S47, S75, S107, S108, S185	S23, S43, S173,	S10, S30, S109, S184, S194	S20, S33, S72, S73, S86, S134, S175
Indonesia (9)	S80, S88, S92, S205	S82	S143	S124	S90	S166
Thailand (6)	S39, S64, S112		S63	S165	S135	
South Korea (6)			S121	S54, S91, S120	S52, S172	
Italy (5)	S24, S103			S113	S110	S94
Bangladesh (5)			S152, S106	S68, S169	S22	
Canada (5)	S139			S44, S83, S132,	S119	
Taiwan (5)	S21, S59, S126	S35				S13
United Kingdom (4)	S104	S159			S79	S9
Turkey (4)	S95	S118	S74			S136
Australia (4)	S77, S182			S215		S12
Brazil (3)	S168			S1, S85		
France (3)				S2	S179	S120
Japan (3)		S128		S3	S195	
Philippines (3)				S147, S153, S204		
Singapore (3)	S56			S7, S51		
Egypt (2)					S174	S214
Greece (2)	S70					S115
Mexico (2)	S89			S100		
Pakistan (2)	S14					S87
Sweden (2)				S180	S84	
Vietnam (2)				S36, S105		
Austria (1)				S96		
Belgium (1)	S202					
Denmark (1)					S131	
Equator (1)	S206					
Ethiopia (1)	S197					
Georgia (1)				S190		
Germany (1)					S19	
Ghana (1)					S167	
Honduras (1)	S140					
Iran (1)				S200		
Ireland (1)	S40					
Malaysia (1)						S67
New Zealand (1)	S150					
Nigeria (1)						S18
Oman (1)	S25					
Romania (1)				S188		
Russia (1)	S218					
Saudi Arabia (1)		S193				
Spain (1)				S78		
Tanzania (1)						S42
Tunisia (1)		S65				
Ukraine (1)						S118
